# COVID 19 pandemic challenges and their management: A review of medicines, vaccines, patents and clinical trials with emphasis on psychological health issues

**DOI:** 10.1016/j.jsps.2022.05.004

**Published:** 2022-05-24

**Authors:** Sradhanjali Mohapatra, P. Ayash Kumar, Uzma Farooq, Pooja Jain, Rahmuddin Khan, Nazia Hassan, Athar Shamim, Mohammad Javed Ansari, Ahmed S. Alalaiwe, Mohammed F. Aldawsari, Mohd Aamir Mirza, Zeenat Iqbal

**Affiliations:** a Nanotechnology Lab, School of Pharmaceutics Education and Research (SPER), Jamia Hamdard, New Delhi, India; b Sun Pharmaceutical Industry Limited, R& D Centre, Gurugram, India; c Department of Pharmaceutics, College of Pharmacy, Prince Sattam Bin Abdulaziz University, Alkharj, Saudi Arabia

**Keywords:** SARS‐CoV‐2, COVID 19, Physical health, Mental health, Medication, Vaccine, Clinical trial, Patents

## Abstract

The SARS‐CoV‐2 (COVID 19) paroxysm is a dominant health exigency that caused significant distress, affecting physical and mental health. Increased mortality, a stressed healthcare system, financial crisis, isolation, and new living and working styles enhanced societal commiseration leading to poor health outcomes. Though people try to maintain good physical health but unfortunately the mental affliction is still ignored. Poor psychological health has emerged as a burgeoning social issue and demands attention. Henceforth, the fundamental objective of this review article is to collate information about COVID-linked physical and psychological agony in diverse population groups with related symptoms and accessible diagnosis techniques. Recent studies have unraveled the fragile mental states of people who have either contracted COVID 19 or had near and dear ones falling prey to it. The impact of the epidemic on the human mind both in short and long-term, with possible risk and preventive factors together with suggested solutions for maintaining good health have also been discussed here. It also enlists the available medications, vaccines and investigational research in the form of patents and clinical trials. This article can be taken as an updated information sheet for COVID 19, accompanied by its management techniques with special emphasis on coping strategies for mental health. Further, it may also assist the policymakers to devise approaches that could enable the public to overcome the pandemic-driven adversity not only in the given situation but also futuristically.

## 1 Introduction

The COVID 19 pandemic is a major health exigency that has caused significant distress and deteriorated the quality of life (QoL) of individuals. While causing numerous fatalities across the globe and leaving many impacted by long covid effects, this pandemic has affected society in myriad ways. Although most recuperated well, its fierce emergence has left the healthcare system in shambles and ushered the world economy into the doldrums. The shutting down of small-time businesses and activities related to infrastructure has added to the rising rates of unemployment and financial insecurities posing unprecedented challenges for the entire population. One thing which is severely depreciated by this pandemic is the health of the individual which entails both physical as well as mental health. “WHO” defines health as a state of complete physical, mental and social well-being and not merely the absence of disease or infirmity. It simply means good health can be achieved by maintaining both physical as well as mental health (WHO, WHO, n.d., WHO, n.d). Additionally, emotional, spiritual, and financial well-being also contribute to the individual's general health status as these are linked to stress levels. It is apparent that this deadly virus disturbs our physical health which manifests as diverse symptoms in an infected individual whereas mental health is not outwardly noticeable, demanding special attention (Salari et al., 2020).

Good physical health is prerequisite for optimum functioning of individuals, is directly related to their overall productivity, and is the index of 'one's well-being. Any compromises thereof would reduce the 'body's inherent capacity to fight infections and may invariably lead to poor mental health. Henceforth, consuming a nutritious diet, following a healthy lifestyle, with regular physical exercise and proper sleep remains the key to and uphold good physical health. Unfortunately, the forced isolation and physico-social disconnect due to the current COVID 19 pandemic has disrupted our daily routine with adverse physical health outcomes. The Pan Global nationwide lockdowns have become the mainstay governmental tool to restrict and slow down the spread of the virus, leaving no means to cater to personal health and consequently waning off public health.

The unforeseen societal changes have resulted in an emergence of “fear of unknown” which is collectively perceived by the populace and expectably so. The primary contributors to these negative connotations include the birth of newer life realities like facing the temporary unemployment, working indoor, home-education for children, exploitation of virtual platforms, social distancing and lack of physical contact with friends and colleagues. The conjoined effect of the “pandemic stress” and “poor physical health” is causing an imbalance to the mental well-being. The situation could be scarier for those who previously suffer from mental debilitation of any kind. It has been noted that the impact on the mental health has been felt throughout the affected countries although it is much accentuated in developing countries. Therefore, it is essential to enumerate the effect of COVID 19 on stress to better deal with this situation.

COVID 19 is causing disaster worldwide, but people of developing countries are getting more frightened, unsecured emotionally, depressed, and in a stage of confusion. Most of them are unaware about symptoms of COVID 19, its preventive measures and management techniques. Sometimes they consider the infection as social stigma primarily due to lack of knowledge, which deteriorates social stigma primarily due to lack of knowledge, which deteriorates the condition. Even if the government has taken necessary steps and strict measures, people's awareness of infection remains the most significant factor in restricting the prevalence of diseases. General knowledge and understanding about the corona virus, causes of infection, symptoms, diagnostic techniques, accessible mode of treatment, and vaccines are essential to fight against the infection. Simultaneously it is important to track the investigational research such as patent and clinical trials to have an upgraded knowledge about the ongoing investigation (Singh et al., 2020, Ahmed et al., 2020, Alahdal et al., 2020, Mohapatra et al., 2021).

“Good mental health is absolutely fundamental to overall health and well-being”. In this context, the current review is an attempt to explore and update the physical and mental health status of various populaces such as healthcare professionals, elderly, children, adolescents and pregnant women, etc. for such rapidly spreading contagious illness and their management procedures. It compiles various symptoms related to physical health and enlists several risk factors for mental health issues amid COVID 19 pandemic. Further, it tries to collate the different patent, clinical trials related to COVID 19 and enlists available medications and vaccines to fight against this virus. This article discusses many challenges and suggested solutions, together with the authors’ views. Lastly, it may act as a platform to understand the psychiatric aspects of the COVID 19 and advocates a case for the serious intervention of psychiatrists who may propose using effective medications and other techniques for this unattended health issue.

## 2 Physical health

Physical health may be defined as “the ability to perform daily tasks and live comfortably, free from disease or disability. A healthy body is a reflection of a healthy mind and vice versa that leads to an improved QoL. Regular exercise, balanced diet, and adequate rest contribute to good health. Following a healthful lifestyle is an absolute necessity to maintain good physical health. It decreases the risk of disease by maintaining body composition, activeness, muscular strength and resilience of the individual. The current corona epoch restricts the lives of the people within their home by disturbing their lifestyle and physical health.

Coronavirus disease is a pernicious disease caused owing to the SARS -CoV-2, a diverse family of viruses affecting humans and animals equally. Several types of corona viruses that produce mild to moderate respirational sickness in humans and recover without demanding special treatment. Others, such as SARS-CoV and MERS-CoV, can cause more acute respiratory ailments. A SARS-CoV-2 (COVID 19) is a novel strain of coronaviruses, which can have potentially major complications, such as trouble breathing and pneumonia. Patients infected with SARS-CoV-2 show a wide range of symptoms from asymptomatic cases to acute respiratory distress syndrome and many others as summarized in the Table 1. Although, many have rescued without needing special therapy but older individuals and people having other medical complications (such as chronic respiratory disease, diabetes, cardiovascular disorder and cancer) are more prone to develop critical illness even lose their life (Gasmi et al., 2021). Literature has been well established that patients having co-morbidities are seen to have a high mortality rate after COVID 19 (Bull et al., 2020, Huang et al., 2020a, Wang et al., 2020a, Wang et al., 2020b, Yang et al., 2019). Further, researches have shown the connotation of reduced physical activity and lengthy sedentary behavior with poor health outcomes (Korczak et al., 2017, Chaput et al., 2020, Haapala et al., 2017, Archer and Garcia, 2014).

**Table 1:** Symptoms related to COVID 19.

Common Symptoms	Other symptoms	Emergency Symptoms	Long-term symptoms	Laboratory observations in COVID 19 patients
Fever	Sore throat	Trouble breathing	Psychological problem	Leukopenia
Dry cough	Chills, sometimes with shaking	Constant pain or pressure in chest	Disease linked with heart, liver, kidney, brain, or thrombotic disease besides pulmonary disease	Lymphopenia
Fatigue	Loss of smell & taste	Pneumonia	Risk of bacterial & fungus infections	↑ Levels of aminotransferase
Body aches	Headache	Acute respiratory symptoms		↑ Level of C-reactive protein,
Loss of appetite	Congestion & runny nose	Septic shock		↑ Level of D-dimer
Shortness of breath	Nausea, vomiting & diarrhoea	Exaggerated inflammatory response		↑ Level of ferritin
Mucus or phlegm	Blood clots	Bluish face &lip		↑ Level of lactate dehydrogenase
Tachypnoea	Chest distress	Sudden confusion		

On the other hand, the undisciplined food habits and physical inactiveness may lead to the risk of metabolic disorder (Jiménez-Pavón et al., 2020, Narici et al., 2020) and the development of lifestyle diseases such as obesity. The coexistence of these disease conditions impaired the person's immunologic system, making him more susceptible to infection (Marazuela et al., 2020). Therefore, besides preventive methods (such as regular hand cleaning, using masks, and social distancing), people need to protect their physical wellbeing by doing various physical tasks. Along with exercise, proper nutrition laterally with a balanced diet and adequate rest also help us to be safe in the pandemic. Regular exercise may boost the immune system, modulates inflammation, and controls the viral gateway (Fernández-Lázaro et al., 2020, Khoramipour et al., 2021). Additionally, Yoga; chanting may also help to improve the overall health of the individual (Nagendra, 2020, Jasti et al., 2020, CTRI/, 2020a, Ransing et al., 2020, CTRI/, 2020b). The following Table 2 conscripts various available medications including immunomodulatory drugs and supplements to manage COVID 19. This may act as a resource with all the information organized, which help doctors, pharmacists, and patients have updated treatment regimens.

**Table 2:** List of available medications for COVID 19 treatment.

**Classification**	**Name of the Drug**	**Common Brand names**	**Company name**	**Route of administration**	**Mechanism of Action**	**Side Effects**	**Approving authority for COVID 19**	**Reference**
Anti-Virals	Remdesivir	Veklury	Gilead Sciences	Parenteral	A prodrug of Remdesivir tri phosphate, an adenosine analogue. ↓ RNA-dependent RNA polymerase	↑ hepatic enzymes, hypersensitivity/allergic reactions, nausea & bleeding	FDA & DCGI	(WHO, 2020) (Smith et al., 2020)
		Remwin	Sun Pharma					
		Cipremi	Cipla					
		Covifor	Hetero healthcare ltd					
		Redyx	Dr. Reddys Laboratories					
	Favipiravir	Avigan	Toyama Chemical	Oral	↓ RNA-dependent RNA polymerase thereby ↓ viral RNA synthesis	↑ anti-coagulation	DCGI & FDA	(Munir et al., 2020)
	Lopinavir, Ritonavir (HIV Protease inhibitor)	Kaletra	Abbvie	Oral	By ↓ the main protease enzyme of COVID 19 virus (3CLpro or MPro) by disrupting the viral replication process & its subsequent release from the host cells	Pancreatitis, ↑ in liver enzymes, ↑ blood sugar/ diabetes, heart arrhythmia, ↑ in cholesterol level & insomnia	FDA & DCGI	(Jienchi and Kome, 2020) (Abrams et al., 2000, Jienchi and Kome, 2020, Abrams et al., 2000)
		Aluvia	Abbott					
		Lopimune	Cipla					
		Emletra	Emcure pharma					
		Hivus LR	Aurobindo					
		Ritocom	Hetero					
Anti-Parasites^##^	Ivermectin	Ivermectol	Sun Pharma	Oral	By ↓ of host importin (α/β1) nuclear transport protein, a key transport process that has been controlled by virus during infection by ↓ the host’s response to antivirus	Dizziness, pruritis & nausea	WHO & DCGI	(NIH, 2020, Nih, 2021, Caly et al., 2020)
		Ascapil	Abbott					
		Ivoral Forte	Cadila Healthcare					
		Iverdis	Bionova					
Anti-Protozoal^#^	Chloroquine	Nivaquine	Abbott	Oral	By ↓ of viral RNA polymerase & ↓ of ACE2 receptors	Loss of appetite, diarrhoea, risk of retinal damage & seizures	WHO, FDA & DCGI	(Vincent et al., 2005)
		Lariago	IPCA					
		Resochin	Bayer					
	Hydroxy chloroquine	Hydroquin	Sun Pharma	Oral			WHO, FDA & DCGI	(Abella et al., 2021, Klimke et al., 2020, Mitjà et al., 2021)
		HCQS	IPCA					
		RHQ	Abbott					
Anti-Bacterials	Azithromycin	Azro	Abbott	Oral	↓ the inflammatory reactions & ↓ excessive cytokine production during viral infestation thereby ↓ of mucous hyper secretion & hence ↓ the production of reactive oxygen	Abdominal pain, stomach upset & tiredness	WHO, FDA & DCGI	(Butler et al., 2021, Echeverría-Esnal et al., 2021)
		Zetorin	Svizera health care					
		Trulimax	pfizer					
		Zithromax	pfizer					
		Azithral	Alembic Pharmaceuticals Ltd					
		Azax	Sun Pharma					
Immunomodulators	Dexamethasone	Wymesone	Wyeth Ltd	Oral & Parenteral	↓ the enzyme phospholipase A2 & blocks the synthesis of the inflammatory mediators & counters the body’s ↑ inflammatory response	↑ in appetite, mood changes, agitation & headache	WHO, FDA & DCGI	(Ahmed and Hassan, 2020)
		Dexona	Zydus cadila					
		Demisone Inj	Cadila Pharmaceuticals					
		Decamycin Inj	Sun Pharma					
		Intensol	West-Ward					
	Glucocorticoids such as Prednisone, Methyl prednisolone, Hydrocortisone	Prednisone		Oral	Prednisone and Methyl Prednisolone: intermediate acting, used for management of system inflammatory response in severe covid 19 infection that lead to injury to lungs & multiple organ dysfunction Hydrocortisone: short acting, control septic shock in COVID 19 management	Prednisone: Headache, dizziness, puffiness in face, blurred vision & weight gain Methyl Prednisolone: Stomach upset, vomiting, restlessness, acne Hydrocortisone: Headache, dizziness, muscle ache, swollen ankles & indigestion	WHO, FDA & DCGI	(Bozzette et al., 1990, Sterne et al., 2020; “Centers for Disease Control and Prevention. Parasites - strongyloides: resources for health professionals. 2020;,” n.d.; Stauffer et al., 2020)
		Rayos	Harizon Pharma					
		Methyl Prednisolone		Oral			WHO, FDA & DCGI	
		Medrol	Pfizer					
		Predmet	Sun pharma					
		Hydrocortisone		Oral & parenteral			WHO, FDA & DCGI	
		Primacort Inj	Macleods pharmaceuticals Ltd					
		Hisone	Samarth Lifesciences pvt Ltd					
	Colchicine	Zycolchin tablets	Zydus Cadila	Oral	Mitigates the inflammation related manifestation by ↓ the movement of neutrophils & ↓ the signalling of inflammasomes of cytokines (Interleukin-1)	Diarrhoea, vomiting, stomach pain,muscle cramping & loss of appetite with occasional side effects such as meuromyo toxicity & blood dyscrasias	WHO, FDA & DCGI	(Reyes et al., 2021, NCT0447, 2020, Indraratna et al., 2018)
		Colcrys	Takeda Pharmaceuticals					
		Mitigare	Hikma Pharmaceuticals					
		Gloperba	Avion Pharmaceuticals					
	Fluvoxamine	Fluvoxin	Sun Pharma	Oral	↓ production of inflammatory cytokines	Diarrhoea & Nausea	WHO, FDA & DCGI	(Rafiee et al., 2016, Lenze et al., 2020, Seftel and Boulware, 2021)
		Revilife	Abbott					
	Immunoglobulins: α, β Interferones	Interferon α-2b		Parenteral	Interferon α-2b & β-1a are the cytokines with anti-viral properties & also ↓inflammation	Interferon α-2b: Dizziness, blurred vision & insomnia Interferon β-2a: Loss of appetite, FLU like symptoms & fatigue	WHO, FDA & DCGI	(Winthrop et al., 2018)
		Zavinex	Zudus Cadila					
		Egliton	Sun Pharma					
		Interferon β-2a						
		Rebif	Merck					
		Betaferon	Zydus Cadila					
	Interlekin (IL-6) Inhibitors: Taclizumab	Actemra	Roche	Parenteral	↓ the release of IL-6 in bronchial cells during COVID infections thus ↓ the ↑ cytokines release during COVID linked systemic inflammation & leads to hypoxic respiratory damage	Cough, sore throat & hypercholesterolaemia	FDA & DCGI	(Marchese et al., 2020, Le et al., 2018, Interleukin-6 Receptor Antagonists in Critically Ill Patients with Covid-19, 2021, Rosas et al., 2021)
	Kinase Inhibitors: Barcitinib in combination with Remdesivir	Oluminant	Eli Lily	Oral	Interference with phosphorylation of STAT proteins leading to immune system activation	Respiratory tract & urinary tract Infection	DCGI & FDA	(“Fact Sheet for Patients, Parents and Caregivers Emergency Use Authorization (EUA) of Baricitinib,” n.d.; Richardson et al., 2020, Food and Drug Administration)
		Barinat	Natco Pharma Ltd					
	Ruxolitinib (Janus kinase inhibitor esp JAK1 & JAK2)	Jakavi	Novartis	Oral	Modulate downstream inflammatory responses by interfering with phosphorylation of STAT protein	Skin rashes, nausea & burning during urination	FDA	(Cao et al., 2020)
Monoclonal Antibodies	Neutralising Monoclonal antibody: Bamlanivimab + Etesevimab Recombinant Human monoclonal antibody: Casirivimab + Imdevimab	Bamlanivimab Etesevimab Regen-cob (Casirivimab + Imdevimab)	AbCellerra and Eli Lilly Eli Lilly Regeneron	Parenteral	Bamlanivimab: target the receptor binding site of spike protein Etesevimab: bind to overlapping epitome in receptor binding domain of spiked protein Casirivimab & Imdevimab: bind to non-overlapping epitome of spike protein Bind of these to spike protein prevents the COVID Infection & ↓ the progression of disease	Nausea, headache, pruritis & dizziness	WHO, FDA & DCGI	(Dougan M, Nirula A, Gottlieb RL, n.d.; Administration., n.d.; “Food and Drug Administration. Frequently asked questions on the emergency use authorization of casirivimab + imdevimab.2020.,” n.d.)
			
Supplements	Vitamin C	Limcee	Abbott	Oral	A free radical scavenger having anti-inflammatory actions & ↑ cellular immunity	Although safe but mega dose can cause heart burns & abdominal cramps	WHO, FDA & DCGI	(Wei, 2020, Thomas et al., 2021, Fowler et al., 2020)
	Zinc	Mostly administered with vitamins		Oral	Impair RNA replication	High dose ↑ cytotoxicity	WHO, FDA & DCGI	(Abd-Elsalam et al., 2020, Thomas et al., 2021, Fact sheet for healthcare providers, Fact Sheet for Patients, National Institutes of Health. Office of Dietary Supplements, 2020)
	Vitamin D	Vitamin D plus	Baksons Homeopathy	Oral	Modulate immune responses by activating Vit D metabolites	High dose can cause fatigue, dry mouth, anorexia, & metallic taste	WHO, FDA & DCGI	(Martineau et al., 2017, Murai et al., 2021)
		Calcitas	INTAS					
Blood thinners	Heparin	Beparine	Bio E	Parenteral	↓ micro-thrombus development by blocking uncontrolled blood coagulation & hence ↓ the risk of major organ failure	Skin warmth or discoloration(specifically on hands & feet), shortness of breath, dizziness, anxiety, sweating, loss of appetite, nausea & vomiting	WHO, FDA & DCGI	(Hippensteel et al., 2020, Buijsers et al., 2020, Gozzo et al., 2020, Tang et al., 2020)
		Bioclot	Elfin					
		Lupenox	Lupin					
		Nuparin	Troikaa Pharmaceuticals ltd					
Convalescent Plasma (Currently administration of this is on hold)	Plasma containing antibodies (from person recovered from COVID 19) to Corona virus	–	–	–	Supressing the virus & inflammatory response	Transfusion-transmitted diseases such as hepatitis B, C, HIV, hypothermia, allergic & febrile nonhemolytic reactions	WHO, FDA & DCGI	(Hueso et al., 2020, Fung et al., 2021)

# No more in use, amid serious concerns about the drug’s safety.

## No more recommended due to lower efficacy on Covid 19.

WHO: World health Organisation.

FDA: U.S. Food and Drug Administration.

DCGI: Drugs Controller General of India.

**Fig. 1:**
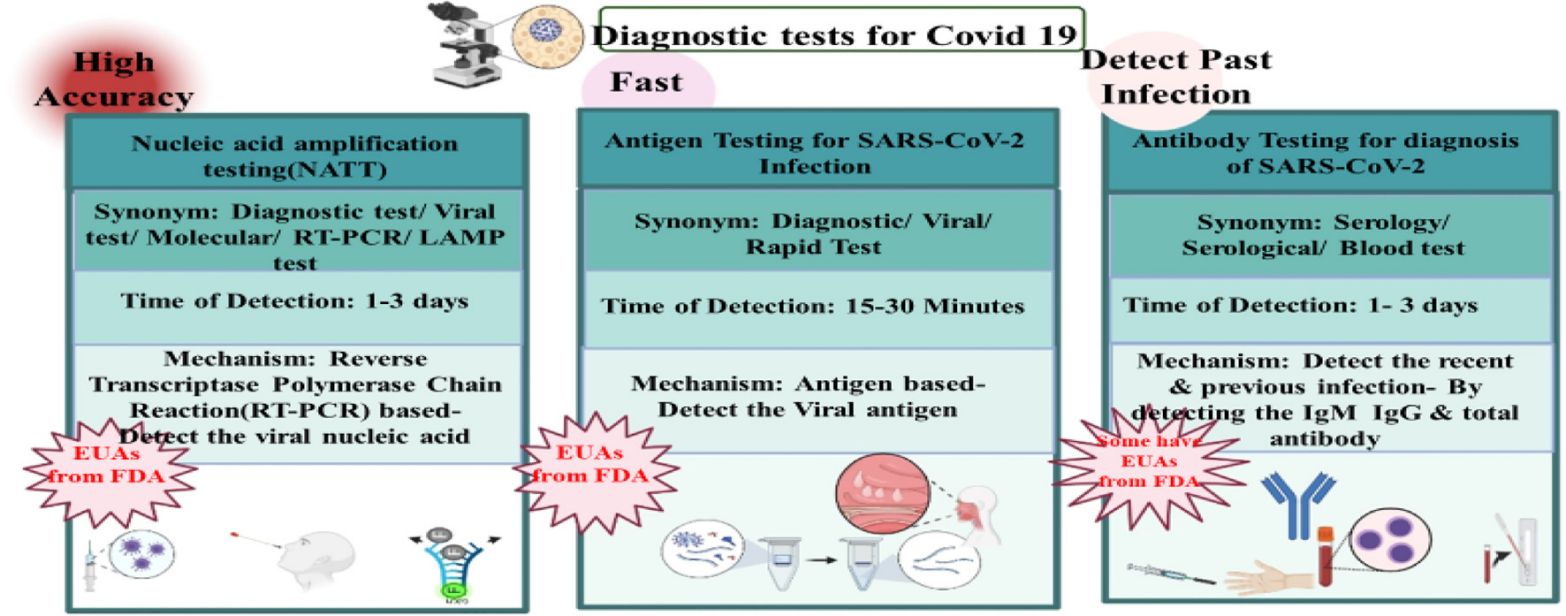
Various diagnostic tests for COVID 19.

Additionally, timely diagnosis and earliest therapy of COVID 19 are the key steps for superior management of the infection and is essential to reduce the fatality ratio in COVID 19 patients. Ignoring the symptoms at the initial stage and deviating the public health protocol are found to be the vital reason for spread of infection and depreciating public health; as reinforcing immunity at the initial period are easy rather than severe stages of infection. So it is essential to overcome ignorance with adequate knowledge and awareness which will be valuable to boost the febrile patients with cough to seek immediate doctor’s consultation (Huang et al., 2020b, Peng et al., 2020, Peng et al., 2020). Fig. 1 illustrates various diagnostic tests for COVID 19.

## 3 Mental health

**Fig. 2:**
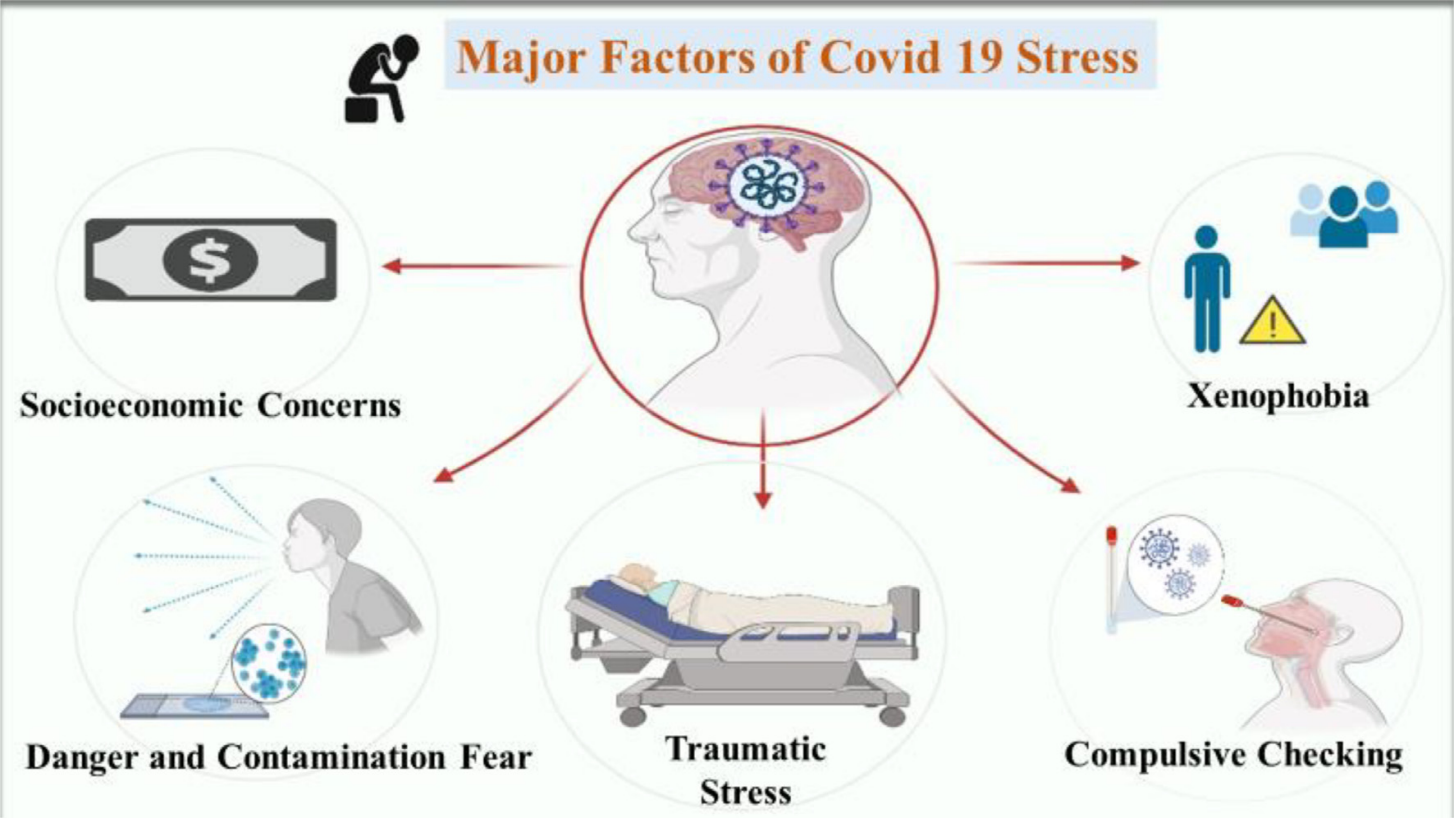
Major risk factors for COVID 19.

Mental health describes the emotional and psychological well-being that helps to lead a relatively happy and healthy life. When we go through a difficult time and facing challenges it is obvious to receive negative emotions. Even though all the individuals are different from each other, there are certain factors that will affect our mental health. Different life experiences, upbringing conditions, environment and genetic factors can influence our thinking and response to challenges and opportunities. There’s no single “right way” to react, so everyone may react differently to different events. The coronavirus (COVID 19) outbreak may cause a diverse negative impact (summarized in Table 3) that might have different contributing factors summarized in the following Fig. 2.

**Table 3:** COVID 19 related psychological distress (Coping with Stress, 2019, Guo et al., 2020, Ni et al., 2020, Liu et al., 2020).

Feelings	Reason
Negative emotions	Fear, worry, anger, sadness, numbness, or frustration
Fear of becoming infected	Restriction to go to hospital/ health care facilities & other places
Fear of illness & death	Contributed to the novelty of the illness and associated uncertainty in its treatment
Feeling of helplessness	Unable to protect the family from the deadly influence of this virus
Fear associated with the past experience	From earlier epidemic
Fear of losing their income	Unable to work through isolation or due to economic recession
Fear that quarantine	Socially isolation from friends & family
Depression, helplessness, boredom & loneliness	Lack that kind of comfort and support during isolation
Difficulty sleeping	Negative thoughts & nightmares
Physical agony affecting negative feelings	Headache, body ache, stomach distress, loss of smell & rashes on skin etc.
↑ urge for alcohol, tobacco & other narcotics	Depression

As a consequence of the emergence of corona virus disease public health actions, such as quarantine, social distancing, lockdown are taken into contemplation to reduce the spread, but at the same time, it also imposed persons to feel isolated and lonely and leads to the intensification of stress, anxiety and depression (Rogers et al., 2020, Wang et al., 2021a, Tee et al., 2020a, Tee et al., 2020b). Several studies have been carried out to find the consequence of quarantine on the individual's psychological status, and the results show a much greater occurrence of psychological distress amongst the quarantined population than those who did not. So this period seems to have important and dysfunctional psychological stress on the individual’s mental health, causing anxiety, depression, anger, and stress-related disorders both in the short-term and long-term period which further worsening their overall health status (Henssler et al., 2021, Bai et al., 2004, Reynolds et al., 2008, Sprang and Silman, 2013, Hawryluck et al., 2004, R., et al., 2003, Hull et al., 2005). Table 4 conscripts some of the potential risk and protective factors underlying mental distress in response to the COVID 19 lockdown. The risk factors help to better recognize the reason why certain people are more predisposed to severe illness, whereas the protective factors give methods to deal with the situation with necessary precautions.

**Table 4:** Risk and protective factors for the mental distress during the COVID 19 lockdown (Sun et al., 2020, Novotný et al., 2020, Browning et al., 2021, Ozamiz-Etxebarria et al., 2020, Jayakumar et al., 2020, Ajduković et al., 2021; C. Wang et al., 2020; C. Wang et al., 2020; Wang et al., 2021b, Renaud-Charest et al., 2021, Wang et al., 2021a; C. A. Tee et al., 2020).

Risk factors	Protective factors
Age (<30 years)	Practicing hand hygiene measures & wearing mask
Gender: Females	
Race or ethnicity	Limited time on health info/statistics
Socio-economic status	Confidence in clinicians identifying COVID 19
Strong education background & lack of formal education	
Feeling of loneliness (experiencing quarantine)	Staying with children
Lifestyle components	Spending two or more hours in the open air everyday (with all precautions)
Unemployment/retired/self-business/ employed in private sector	Psychological resilience
Discrimination by other countries/ Xenophobia (fear and hatred of strangers or foreigners)	High perceived likelihood of surviving COVID 19
Illness perception	Active coping style
Occupational exposure risks	Use of face Mask
Having confirmed/suspected infection or having a relative with confirmed/suspected infection	
Living with family members or a partner	
Media exposure	
Smoking	
Amount of screen time spend by the students on each day	
Parenthood (>2 kids)	
Passive coping style	
Patients with positive previous psychiatric diagnoses/ Depression severity	
Low confidence in medical service/ system	
Presence of chronic diseases	
Physical symptoms resembling COVID 19 infection	

One of the recent investigations indicates that lack of social connection is linked with a variety of deprived physical and mental health consequences, even early mortality. Therefore it is an undeniable fact that physical distancing carried out to limit the spread of the corona virus has a potential negative impact on these health outcomes affecting life severely (Morina et al., 2021)According to other studies, the COVID 19 contagion increases the psychological health problems in COVID 19 patients and quarantined persons but also in health care workers and patients with non-infectious chronic disease, demanding urgent interventions of mental health management measures (Wu et al., 2021, Pappa et al., 2020, Nochaiwong et al., 2020, Kumar, 2019, Kumar et al., 2020). Another study carried out in the Indian population concluded that students and health professionals shows higher psychological distress than others and necessitate special attention as they are the main stakeholders in society (Rehman et al., 2021). A further investigation conducted to check the mental health condition of pregnant females during the COVID 19 plague and concluded that pregnant ladies are more prevalent to anxiety and depression during this period, particularly younger pregnant women (Fan et al., 2021).

Evidence has been found that people having mental illness are at higher affliction of getting infected with COVID-19 (Mazereel et al., 2021). If they had any previous history of mental disorder then the relative risk of having infection is little more (Taquet et al., 2021). Hence, taking into account the higher risk for COVID 19, it has been advised that persons with mental illness should be vaccinated on a priority basis. Yet, the patients with mental diseases are reluctant to get vaccines for COVID-19. However, there is limited literature on the rates of vaccine reluctancy among people having mental illness. Further, in case of depression and schizophrenia the immune response to vaccines get reduced leading to inadequate production of antibodies, which may not give enough protection to the patient even after vaccination. Additionally, there are rising fears for hesitancy in the general public because of associated side effects and doubt about the efficiency (Khubchandani et al., 2021, Razai et al., 2021, Shacham et al., 2021). All these above experiences are factual in the face of this significant challenge of the COVID 19 pandemic. Concern for one's own and one's loved ones, rapid modifications in one's way of life (e.g., education, employment, social get-togethers), interrupted plans caused by travel restrictions, social isolation, and quarantine are some of the population's new-fangled challenges. Thus it’s essential to recognize the seriousness of the situation. At this point, the authors want to highlight that the emergence of psychiatric conditions and mental health were identified as the 10th most frequently investigated subject in the course of the COVID 19 pandemic (Tran et al., 2020). Thus to understand and accept the fact we have to first analyze the mental status of the individual population with their mindset for such pandemic, which is imperative to meet the global crisis. A startling observation in regard with the covid impact is its potential to exacerbate health issues amongst vulnerable groups like mental health sufferers, geriatric population, pregnant women and even less privileged unemployed members of the community (Holmes et al., 2020, Power et al., 2020, Oecd, 2020). This emergence warrants attention and indicates the collation and discussion of the COVID −19 data related to psychological stress in the various population (enlisted in the Table 5). Furthermore, concerted efforts towards accumulation of real world evidence (RWE) with a focus on their possible management procedures, is highly desirable. This repository of information will act as a resource for the patients, clinicians, regulators, and policymakers who would be better equipped to deal with the present scenario of crisis and any future pandemics.

**Table 5:** Population at risk for mental health issues during the COVID 19 outbreak (Torjesen, 2020, Hosgelen and Alptekin, 2021; WHO, n.d.; WHO, n.d.; Khadse et al., 2020).

S. No.	Healthcare workers
1	Police personnel (Specially in countries like India)
2	Children and adolescents
3	Older persons
4	Pregnant women
5	Patients suffering from COVID 19
6	Persons at domestic abuse risk
7	Individuals who have had previous history of mental sickness
8	Women, in particular, if they have to juggle home-schooling, working from home, and do the household tasks
9	Individuals struggling financially or in lower socioeconomic

The following section elucidates the psychological agony in diverse population groups along with suggested solutions.

### 3.1 Healthcare professionals

The COVID 19 outbreak 2019 evolved to be one of the central health crises affecting people of all continents, realms, races, and socioeconomic groups and posed an increasing challenge for healthcare professionals who are designated as the front-line workers (Chen et al., 2020a, Shi et al., 2020). Understandably, in this pandemic, health professionals are experiencing the same situations as other individuals but are burdened with insurmountable mental and physical stress (Petzold et al., 2020). In the perspective of any epidemic, health workers are expected to have long working hours without availing any leave. Massive workload, an growing figure of infected cases, rise in the death rate, risk of being infected while treating patients, lack of any specific medication, extensive social media reporting, shortage of personal protective equipment, lack of preparedness and every day’s new challenges can contribute to various psychological and mental illnesses (Nct04497415, 2020, Lai et al., 2020). Further morals and medical ethics that emphasize self-sacrifice as a vital principle of this noble profession, may add to job pressure. According to past reports, health care workers having a high risk of contamination have persistent stress and higher degrees of anxiety and depression (McAlonan et al., 2007). The study carried out to investigate stress reactions among staff members in a hospital involved in the outbreak of severe acute respiratory syndrome (SARS) located in east Taiwan concluded that 5% of this group suffered from an acute stress disorder, 20 % felt stigmatized and debarred in their neighborhood since they involved in hospital job and 9% reported reluctance to work (Bai et al., 2004). Research on the occurrence of anxiety, depression, stress and restlessness during the COVID 19 eruption among healthcare workers suggested that a significant percentage of them experienced mood and sleep problems in this situation. Additionally, female healthcare staff, nurses, younger medical staff, and workers in this area have shown higher rates of emotional symptoms than, than males and other counterparts (Pappa et al., 2020, Vizheh et al., 2020). Another investigation on health care staff in hospitals in Wuhan and other parts in China described the mental problem, mainly in nurses, females, and front-line healthcare workers who are involved directly in the diagnosis, management, and patients care during COVID 19 situation (Lai et al., 2020, Sun et al., 2020).

#### 3.1.1 Management

Appreciation by the supervisors, public and patients is one of the powerful motivational tools for reducing stress in health care workers. It helps to boost the kindness and sacrifices of health workers who risk their lives to serve the infected patients. Such gestures may support overcoming empathetic distress and fear to provide care under challenging clinical conditions of the COVID 19 pandemic. Various stress management methods such as training and educational programs may be beneficial for this outbreak (Chua et al., 2004). Personal coping methods such as positive thinking, problem-solving, seeking social support, along with institutional measures such as self-care, infection control, safety, staff support and gratitude, and clear communication are helpful measures for managing stress (Chew et al., 2020). It is advisable to the head, managers, and policymakers to implement supportive, protective, encouraging & motivational, training, and educational interventions, at a regular interval to reduce the psychological stress in front-line workers (Vizheh et al., 2020).

### 3.2 Police personnel

Similar to health care personnel, police are also listed as the “corona warriors” and acted as front-line workers in this pandemic. During this epidemic outburst in populous countries like India, the primary responsibility of the police personnel is to implement the national lockdown and safeguard physical distancing by enforcing laws (Epidemic Disease Act, 1987, and the Disaster Management Act, 2005) along with other social responsibility apart of their regular work profile. This can result in a greater possibility of developing a range of psychological, emotional and perturbation amidst this group. There is adequate prove to document the intensification of depression, anxiety, drug abuse, and suicide among this population (Grover et al., 2020, Di Nota et al., 2020, Edwards and Kotera, 2020). It is evident from the past research that organizational culture and workload are the main issues for boosting stress in police personnel. Stress concerning to job among the police personnel may be attributed to nearly similar factors as discussed above, the reason being participation as front-line workers in the COVID 19 outbreak. Further, susceptibility in female officers is comparatively more than their male counterpart (Collins and Gibbs, 2003, Grover et al., 2020).

#### 3.2.1 Management

Spreading awareness among the staff about mental health complications, conducting training and motivational sessions to develop constructive coping skills, and building resilience can alleviate mental distress. Teaching relaxation practices such as yoga or meditation can also help them to cope with stress positively. Regular physical exercise can also solve the purpose along with special appreciation and support. Regular communication (online medium) with the family members is an effective way to strengthen their support system, especially those who work away from their homes or undergo quarantine. Additionally, persons with a previous history of mental disorder should pay special attention to timely and reasonable consultation (Khadse et al., 2020).

### 3.3 Children and adolescents

COVID 19 contagion is causing interruptions in the universal social structure leading to socioemotional complications. Many countries maintain social distancing and observe the quarantine with government-imposed mandatory restrictions on many outside activities. As a result of which children and adolescents have had their schools closed, and outdoor home activity and social interactions abridged many fold. These restrictions limit this group's physical movement, which further leads to irritability, lethargy, anxiety, depression, stress, and inattention and fear of infection in them (Nearchou et al., 2020, Meherali et al., 2021). Such public restrictions promote stress in this youth population and in their parents and can become a barrier in the normal growth and development of children (Araújo et al., 2020) and may intensify as this isolation continues. Additionally, poor diet, confinement to a place, and increased screen time may cause cases of obesity, eyesight complications, stroke, heart attack and many other ailments (Spitzer, 2021). The declining QoL of young children cannot be ignored and demands special attention as they are the main stakeholders of any country. There is sufficient evidence of the correlation between physical activity and psychological status in the literature(Saxena et al., 2005, Sagatun et al., 2007, Ahn and Fedewa, 2011, Okuyama et al., 2021). Study outcomes concluded that the COVID 19 pandemic is responsible for the new onset of psychological problems among children. The current scenario has taken a heavy toll on differently-abled children. Autstics and ADHD sufferers have emerged as a highly vulnerable group whose mental health deterioration could be detrimental for their future (Panda et al., 2021).

#### 3.3.1 Management

This epidemic outbreak demands immediate action by preparing novel strategies for early psychological interventions to decrease its influence on the mental health of the youth. There is an utmost emergency for establishing an on demand clinical support facility for better amelioration of these problems. The distress quotient among these populations can be reduced by maintaining social networking with friends and relatives, positive thinking, physical exercise and avoiding false social media information. Online resources such as information about psychological health and preventive measures, video counselling, tele-psychiatry and telemedicine services, can be useful to lessen the imaginary fears and ultimately psychosocial stress (Deolmi and Pisani, 2020, Loades et al., 2020, Prisco et al., 2020). It is advisable to the psychologist, doctors, and policymakers to develop new and innovative policies that help them compete with this corona epidemic-driven adversity and ensure normal physical and mental development in children and adolescents(Araújo et al., 2020).

### 3.4 Geriatrics persons

Although people of all eons are affected by the rage of corona, geriatrics are more prone to the negative consequences, which results in decreased QoL and poor mental health. The rapid rate of geriatric mortality in the COVID 19 outbreak may be attributed to compromised immunity, pre-existing ailments such as diabetes, hypertension, thyroid, cardiovascular, menopause, and other chronic conditions that make them more susceptible to viral infections (Sepúlveda-Loyola et al., 2020; *Nanomedicinal Approaches Towards Cardiovascular* Disease, 2021). Usually, older adults suffer from age-related mental stress; a condition which further worsens in this corona age due to restrictions in movement, limited social participation and reduced autonomy. Rapidly growing risk of social isolation, worry and loneliness induced by the pandemic is resulting in various severe short and long-term negative mental health impacts (Bansod et al., 2021). Additionally, QoL has deteriorated and often ends with mortality in this populace. The condition worsens for menopausal females and individuals with a history of psychological illness as they have already strived with poor QoL (Mohapatra et al., 2020; Sradhanjali Mohapatra, 2022).It is reported elsewhere that there is a strong positive relation with social participation and geriatric health (Douglas et al., 2017, Carver et al., 2018, Sepúlveda-Loyola et al., 2020, Perez et al., 2021). Deprivation of social participations of different forms leads to augmented depression, anxiety and cognitive dysfunctions. Nonetheless it is heartening to know that a study conducted on 1,000 U.S. adults claim that though the older population faces stress, anxiety, and depression, but they exhibit better-coping capability with COVID 19 stress in comparison to young adults and have reported to have comparatively less depression and anxiety(“The Older You Are, the Better You May Cope With Pandemic Stress,” n.d.).

#### 3.4.1 Management

Even though there are several recommendations for the elderly to deal with this emerging COVID 19 pandemic, the major management method is to offer adequate emotional support that boosts them to cope with this situation. Family members and caregivers need to be actively and emotionally involved in elderly care focusing more on mental health. Connecting with family and friends over the internet and phone is a better way to ventilate thoughts and helps to deal with isolation. But at the same time, the digital screen time for the news media should be reduced to avoid misinformation and dread statistics. Descriptive news or information with illustrations from authentic sources may help. Maintaining a proper routine, healthy nutrition habits and daily physical activity may improve resilience in older people. Avoidance of self-medication, virtual consultations and telemedicine, tele-psychiatry, proper health education, and psychological counseling could also be beneficial, particularly for individuals having a previous track record of mental ailments(Ahn and Fedewa, 2011, Lebrasseur et al., 2021, Prisco et al., 2020, Gillman-Wells et al., 2021). Further implementing home-delivered settings that enhance mental security may be advantageous in this population (Tegeler et al., 2020). Additionally, stakeholders and policymakers need to take collective action to deal with this challenge ensuring better psychological well-being.

### 3.5 Obstetrics patients

Pregnancy is believed to be a triumphant phase in women’s life but a few are predisposed to negative emotions that lead to psychological distress. Women consecrated with pregnancy are more prone to face psychological agony during their prenatal period. It may increase up to 25% more than the prevailing mental disorder at their reproductive age where the problem is found to be common(Brooks et al., 2020, Howard et al., 2018, Howard et al., 2014, Kendig et al., 2017). Perinatal depression is especially dangerous for women who are carrying a medically high-risk pregnancy (Fairbrother et al. 2017). The pregnancy-induced physiological changes greatly affect the various body system including the immune system of the females. The present COVID −19 era further adds trauma to the condition by exaggerating the damaging effect on this vulnerable population. Although pregnant women have a greater predilection to psychological problems, the COVID 19 outbreak and associated factors further accelerate the condition. Moreover, reduced access to reproductive health services, increased socioeconomic scarcity and social cut-off may worsen the situation. It is well established that expectant females have always been regarded a highly susceptible community. Various findings have been described the vulnerability of expecting females to their emotional insecurity (Stein et al., 2014) and routine trauma (Loomans et al., 2013). It was observed that the pregnancy period during the past pandemic was related with additional adverse clinical outcomes and a higher rate of death (Chui et al., 2004, Mosby et al., 2011). Some investigations conclude that maternal mental health is adversely influenced by the social distancing and impacts of infectious outbreaks. There is an influx of studies stating that the SARS quarantine has led to significant upsurges in levels of anxiety (Dodgson et al., 2010), depression(Linde and Siqueira, 2018) and stress (Davis et al., 2014). Evidences of deteriorating mental health of expectant women and their off springs due to extreme stress conditions, natural disasters or emergencies, have also been reported well(King et al., 2009, Laplante et al., 2004, Cao-Lei et al., 2015). Some of the current studies depict that this pandemic and related factors have negatively affected pregnant women with depression and anxiety and lower quality of mental life and reasonable psychological impact due to isolation(Saccone et al., 2020, Corbett et al., 2020, Basu et al., 2020, Thapa et al., 2020, Ali and Shahil Feroz, 2020, Brooks et al., 2020, Panda, 2021, Zakir et al., 2020). Nevertheless, there is no recent indication for the susceptibility of pregnant women towards COVID 19 illness or those suffering from COVID 19 are more predisposed to develop acute pneumonia, however, compromised immunity may be concluded to be the critical factor(Eastin and Eastin, 2020, Durankuş and Aksu, 2020, Kotabagi et al., 2020, Durankuş and Aksu, 2020, Liu et al., 2021b, Ceulemans et al., 2021, Medina-Jimenez et al., 2020, Berthelot et al., 2020).

#### 3.5.1 Management

Reliable and up-to-date information about the current situation; support, guidance and advice from physician particularly with regards to the prophylaxis and treatment; support and care provided by the family and health care professionals, proper counseling about the potential for severe disease and virtual support; social connectivity; personal hygiene, walking, yoga or exercise are effective methods to deal with this problem. It is imperative to brainstorm and then reach clinical decisions for using approved COVID 19 medicines in expectant mother as the safety of both mother and the foetus is of paramount importance and should ideally be a combined decision between the patient and the clinical team (Gynecologists, 2019, Centers for Disease Control and Prevention, n.d. Considerations for inpatient obstetric healthcare settings., 2020, Society for Maternal-Fetal Medicine, 2020, Rasmussen et al., 2020).

### 3.6 COVID 19 patients

COVID 19 being a highly infectious and contagious ailment with increased incidence of death rate and absence of appropriate treatment gives sufficient reasons to affect patients' mental health. Additionally, socio-emotional and socio-economic factors accentuate the condition. Mental issues in patients may be the sum of both pandemic stress and the physical effects of the disease. Anxiety and depression are the most prevalent symptoms in this population. Furthermore, post-traumatic stress symptoms were surprisingly common after discharge from the hospital(Han et al., 2020, Muruganandam et al., 2020). Nearly 20 % of patients developed a mental health issue while suffering from COVID 19 (Ries, n.d.). There are few studies found in the literature relating to the psychological experience of COVID 19 affected individuals during their hospital stay. According to a survey, anxiety and depression were found to be prevalent in 34.72 percent and 28.47 percent in hospitalized covid patients, respectively (Kong et al., 2020). Another study demonstrated that COVID 19 patients suffer from both physical and mental distress. Further, COVID 19 patients with general pneumonia commonly showed anxiety and depression (Yang et al., 2020). Other investigations conclude that post-traumatic stress symptoms associated with the COVID 19 were observed in the maximum number of clinically stable COVID 19 patients before and after discharge (Bo et al., 2020, Mazza et al., 2020). One *meta*-analysis report specified that the collective prevalence of post-traumatic stress symptoms linked with COVID 19 was found to be 23.88% (Cooke et al., 2020). Further, incidences of confusion and agitation are also reported in ICUs admitted patients having severe COVID 19 infection (Chen et al., 2020b, Helms et al., 2020).

#### 3.6.1 Management

The process of disease treatment for such infection should combine medical therapy along with psychological support. So, medical staff should be trained accordingly. Mental support from medical workers along with physical backing, and regular interaction would keep them motivated to remain positive during the hospital stay (Kong et al., 2020). Traditional counseling may help to mitigate the condition. Mental resources and related amenities must be assigned to support the psychological health problem of the patients. Familiar and adaptive coping skills such as interactions with family and friends, spending time on spiritual things and interaction with mental health professionals in the perspective of this COVID 19 isolation has to be developed (Sahoo et al., 2020, Werner et al., 2020). Quarantine policies and shut-down procedures adapted to avoid the transmission of COVID 19 have obstructed social behaviors in these isolated individuals, so there should be a rethinking for future implementation. It is advisable to initiate cognitive processing therapy for post-traumatic stress disorder and also explicitly use telemedicine, tele-psychiatry during the COVID 19 (Moring et al., 2020). Currently, online psychotherapy such as internet cognitive behavior therapy (ICBT) is proved to be more relevant for dealing mental health particularly during COVID 19 pandemics (Aminoff et al., 2021). It is a modern tool which is emerged with recent technology evolutions and is effective across almost all the geographical area involving several cultures and languages. This is a promising approach to reduce the psychological stress with increased access and better outcomes, in cost effective manner. It has been scientifically proven to be effective in children and adolescents to treat symptoms of depression and anxiety and can be useful for all the above populations to deal with the COVID 19 related psychological agony. (Carpenter et al., 2018, Kumar et al., 2017, Ho et al., 2020, Zhang and Ho, 2017, Liu et al., 2021a, Ying et al., 2021).

## 4 Investigational research

The COVID 19 outbreak has brought about a wide-reaching unprecedented investigation across all countries. Research during a pandemic helps to collect important information that can help to improve outbreak control measures, and catalyses the concerted research efforts in both the clinical settings as well as vaccine development and its trials. Additionally, research into optimizing tools for evaluating health and disease through innovative approaches and technologies could finally lead to improved access to care. It shall also forge ties with the public and pour, benefits to health and disease management organizations. Table 6 enlists several vaccines that are being investigated as well as approved for vaccination against this illness that help to obtain information and comparison among diverse vaccines. Further, it includes some recent patents (collected out of approximately 2,014 published patents) coupled with the prevention and treatment of COVID 19 (“Patent,” n.d.) in Table 7. Additionally, this section enlists (Table 8) some clinical trials from all across the world highlighting the on-going investigations relating to the health (both physical and mental) in relation to COVID 19. A recce of the repository reveals the impetus with which such research endeavors are initiated and executed. It is worth noting that research organizations in developed and developing nations are equally keen to utilize this colossal calamity as an opportunity to unravel their research understanding, which could come in handy in these testing times. It is also evident from the efforts taken by the scientists that they are working towards generating an affordable, accessible and sustainable remedy for this devastating disease.

**Table 6:** List of vaccines approved as well as under investigation for COVID 19 infection.

**Name**	**Innovator/Manufacturer**	**Mode of action**	**Efficacy**	**Side-effects**	**No. of dose**	**Approving authority**	**Reference**
Comirnaty BNT162b2/ Pfizer, BioNTech/ Fosun Pharm vaccine	Pfizer, BioNTech	mRNA vaccine that express the SARS-CoV-2 S antigen, eliciting an immune response	About 95% (clinical trials)	Injection site pain, headache, arthralgia, myalgia, fatigue, chills, pyrexia, injection site swelling or redness, & nausea	2 with 21 to 28 days’ interval	FDA, WHO & EMA	(“pfizer-specific-training_full-deck_27-january-final.pdf e,” n.d.) (“Comirnaty and Pfizer-BioNTech COVID-19 Vaccine,” n.d.)
Moderna vaccine mRNA-1273	Moderna	mRNA vaccine that allows the expression of the SARS‑CoV‑2 spike antigen, provoking an immune response	About 94.1% (phase 3 clinical trials)	Reactogenecity & pain, swelling & erythema at the area of injection, fever, headache, fatigue, myalgia, arthralgia & nausea/vomiting	2 with 30 days interval	FDA, WHO & EMA	(Baden et al., 2021)(“Moderna COVID-19 Vaccine Health Care Provider Fact Sheet,” n.d.) (“COVID-19 ACIP Vaccine Recommendations,” n.d.)
Janssen/JNJ-78436735, Ad26.COV2.S/COVID 19 Janssen / Johnson & Johnson vaccine	Johnson & Johnson	Adenovirus vaccine (Non-replicating) that delivers genes &produce an immune response	About 86% (clinical trial)	Injection site pain, headache, fatigue, myalgia, & nausea Adverse event: thrombosis, thrombocytopenia	Single dose	WHO, FDA & EU	(“Johnson & Johnson’s Janssen COVID-19 Vaccine Overview and Safety,” n.d.)
Covisheild /AZD1222/ AstraZeneca vaccine/ Vaxzevria	Oxford university-Astrazeneca/Serum Institute of India (SII) and Indian Council of Medical Research	Adenovirus vaccine that stabilizes the expressed S-protein by not modifying the coding sequence, leading to release of antibodies & hence ↑immunity	About 79% effective (randomized control trial)	Pain at the injection area, fever, chills, headache, myalgia, fatigue, malaise, arthralgia, diarrhoea, nausea etc. Adverse event: thrombocytopenia and blood clotting	2 doses	WHO & being used in many countries around the globe	(“COVID-19 Vaccine AstraZeneca. (n.d.).,” n.d.) (“Phase III Double-blind, Placebo-controlled Study of AZD1222 for the Prevention of COVID-19 in Adults - Tabular View,” n.d.)(“Safety and efficacy of the ChAdOx1 nCoV-19 vaccine (AZD1222) against SARS-CoV-2,” n.d.)
Sputnik V	Gamaleya Research Institute, Acellena Contract Drug Research & Development in Russia	Adenovirus vaccine that initialize the production of the new coronavirus's covering proteins by providing the coronavirus gene to cells & ↑ the immunity	About 79.4% after 1^st^ dose & 91·6% after2 doses & ↑ the immune system using 2 different formulae even more than using the same version twice & may give longer-lasting protection(in a clinical trial)	Injection site reactions, flu-like illness, headache & asthenia	2 doses with 3 weeks interval	DCGI, India	(“Sputnik V Vaccine. Welcome to Precision Vaccinations. (n.d.).,” n.d.)(“SINGLE DOSE VACCINE, SPUTNIK LIGHT, AUTHORIZED FOR USE IN RUSSIA,” n.d.)
Sinopharm/BBIBP-CorV/ Sinopharm WIBP	Beijing Institute of Biological Products; China National Pharmaceutical Group (Sinopharm)	Inactivated vaccine, that cannot replicate but the presence of spike protein causes an immune response;	About 78.1%	Mild-moderate effects such as headache, fatigue, & pain at injection site	2 doses with 3-4 weeks interval	WHO	(“WHO. (n.d.). Evidence Assessment: Sinopharm/BBIBP COVID-19 vaccine.,” n.d.)(“BBIBP-CorV, Sinopharm COVID-19 vaccine. New Drug Approvals. (2021, March 23).,” n.d.)
CoronaVac	SinoVac Biotech Corporation	Displays its action by introducing killed viral particles to the body's immune system without causing a serious disease response	About 50.7%-62.3% against infections	Pain at the site of injection, headache, tiredness, & myalgia	2 doses with 21 days apart	WHO & China National Medical Products Administration	(“CoronaVac. DrugBank Online. (n.d.).,” n.d.)(“Toscano, C. W. H. O., Araos, R., & Marti, M. (n.d.). Evidence Assessment: Sinovac/CoronaVac COVID-19 vaccine,” n.d.)
ZF2001	Anhui Zhifei Longcom Biopharmaceutial, Institute of Microbiology of the Chinese Academy of Science in China	Recombinant vaccine that uses a COVID 19 spike protein as the antigen against Coronavirus	About 82% against disease of any severity; efficacy was 93% against the α variant & 78% against the δ variant	Well tolerated & immunogenic	3 doses	Uzbekistan & China	(Yang et al., 2021)(Yang et al., 2021)(Yang et al., 2021)
Covaxin (BBV152)	Bharat Biotech, ICMR, NIV, India	Inactivated vaccines do not replicate but able to instruct the immune system to produce a defensive reaction against the infection	About 77.8%, 93.4%, 63.6% efficient against symptomatic, severe symptomatic & protection against asymptomatic COVID-19 respectively	Injection area pain, swelling, redness, & itching accompanied by fever, headache, body ache, nausea, vomiting, weakness, stiffness & malaise	2 doses 4 weeks apart	DCGI & WHO	(“COVAXIN (BBV152) for the Treatment of Covid-19. Clinical trials arena.,” n.d.)
Convidicea/ Ad5-nCoV	CanSinoBIO's adenovirus-based viral vector vaccine technology platform & the Beijing Institute of Biotechnology	Recombinant vaccine (with type 5 vector of adenovirus) that act through heparin-responsive receptor which intermingles with the Ad5 fiber shaft, leading to immunity	About 65.7% (Phase 3 trial)	Pain, fever, fatigue, headache & muscle pain	Single dose	China,	(Cheng et al., 2007)
NVX-CoV2373	Novavax	Stable, prefusion protein nanoparticle	96.4% against the original SARS-CoV-2 (Phase 3 UK trial) & 55.4% against the B.1.351 variant (Phase 2b trial)	Headache, muscle pain, & fatigue	2 doses with 3 weeks apart	WHO	
ZyCoV-D /Zydus' COVID 19 vaccine	Zydus Cadila,	Plasmid DNA vaccine formed the spike protein of the virus & produced an immune response after administration; mediated by the cellular & humoral immunity	66.6 % for symptomatic RT-PCR positive cases	Strong immunogenicity, tolerability & safety in the adaptive Phase I/II clinical trials	3 doses	DCGI	
Corbevax or Biological E’s novel COVID 19 vaccine	Biological E	Recombinant-protein technology, that involves incorporating DNA encoding an antigen to stimulate an immune response in cells	Phase III clinical trial	No data available	2 doses at 28 days interval	CDSCO, India	
BBV154 - Intranasal vaccine	Bharat Biotech	Intranasal replication-deficient chimpanzee adenovirus SARS-CoV-2 vectored vaccine prevent viral illness, by creating an immune response in the nose	Phase II clinical trial	No data available	No data available	Not yet approved	
	Gennova Biopharmaceuticals Limited	mRNA based vaccine	Not reported	Demonstrated safety, immunogenicity, neutralization antibody activity in the rodent & non-human primate models	no report available	Not yet approved	
EpiVacCorona	Vector state Research centre of Virology and Biotechnology in Russia	Peptide vaccine that based on 3 chemically synthesized antigen of the COVID 19 protein attached to a carrier protein & adsorbed on aluminium hydroxide	Overall 79% in Phase-III clinical trial	Severe fever	2 doses	Approved for use in Bealrus, Russia, Turkmenistan	
CoviVac	Chumakov Federal Scientific Center for R&D of Immune & Biological Products	Inactivated, viral vector, egg-based vaccine	Efficacy has not yet been established in a phase III clinical trial	No data available	2 doses, 2 weeks apart	approved for use in Russia	
QazVac	Research Institute for Biological Safety Problems in Kazakhstan	Inactivated vaccine	96% efficacy (in the phase-2)	No serious side effects	2 doses	Approved for use in Kazakhstan & Kyrgyzstan	

WHO: World health Organisation.

FDA: U.S. Food and Drug Administration.

DCGI: Drugs Controller General of India.

EMA: European Medicines Agency.

CDSCO: Central Drugs Standard Control Organisation.

ICMR: Indian Council of Medical Research.

NIV: National Institute of Virology.

**Table 7:** List of patents associated with prevention and treatment of COVID 19.

**Patent No.**	**Title**	**Application**	**Details**
CN111217917B China	Novel coronavirus SARS-CoV-2 vaccine and preparation method thereof	Generate immune response for treating and/or preventing infection of SARS-CoV 2 after immunizing organism	This form of vaccine comprises RBD fusion protein subunit vaccine/ mRNA vaccine/ adenovirus vector vaccine containing RBD fusion protein of the SARS-CoV-2 as core antigen
RU2738081C1 Russia	Peptide immunogens and a vaccine composition against coronavirus infection COVID 19 using peptide immunogens	Used as an constituent of a vaccine against COVID 19 infection	Immunogenic peptides characterized by the amino acid sequence, encompassing antigenic T- and B-cell epitopes of protein S of SARS Cov 2 coronavirus having capability of inducing formation of antibodies, possessing antigen-specific, virus-neutralizing & protective activities
RU2743595C1 Russia	Vaccine composition against COVID 19	A vaccine against COVID 19	Peptide immunogens & a carrier protein, that carry the minimum necessary antigenic determinants for the formation of a specific immune response & induce protective immunity against COVID 19
CN111333704B China	Novel coronavirus COVID 19 vaccine, preparation method and application thereof	Novel coronavirus COVID 19 vaccine	Acquires the dominant antigen (RBD) epitope of the novel coronavirus, then connected with immunoglobulin to prepare RBD-Fc fusion protein, & can be used for developing the protein vaccine of the novel coronavirus COVID 19 & medicaments for preventing/treating the COVID 19
CN111088283B China	mVSV viral vector, viral vector vaccine thereof and mVSV-mediated novel coronary pneumonia vaccine	A viral vector vaccine for coronary pneumonia	Based on mVSV mediation (obtained after multiple modification mutations occur to M protein amino acid site of wild Indiana strain VSV). The mVSV viral vector is embedded or fused with the dominant antigen of the spike protein S of the COVID 19 viral pathogen having better prevention/treatment effect on a new coronary pneumonia virus infected individual
CN110974950B China	Adenovirus vector vaccine for preventing SARS-CoV-2 infection	An adenovirus vector vaccine	Used to prevent SARS-CoV2 infection comprising S protein nucleic acid sequence which is easy to express in human cells & is anticipated as a recombinant virus vaccine for preventing SARS-CoV2 infection
WO2021045836A1WIPO (PCT)	Anti-sars-cov-2-spike glycoprotein antibodies and antigen-binding fragments	An isolated antigen or antibody binding portion	Neutralizing human antigen-binding proteins which bound explicitly to the virus’s spike protein & related methods of using such antibodies & fragments to treat/prevent infections
CN111729079A China	DC vaccine for novel coronavirus, preparation method and application thereof	Dendritic Cells (DC) vaccine aiming at novel coronavirus	Comprises an S protein of COVID 19 & a chemokine CCL 19 having long duration, can quickly recognize antigens, starts the killing function of T cells & generates antibodies
US20200407402A1 United States	Stabilized Coronavirus Spike (S) Protein Immunogens and Related Vaccines	Nanoparticle vaccines	Contain the redesigned soluble S immunogens displayed on self-assembling nanoparticles & also provides olynucleotide sequences encoding the redesigned immunogens. Further providing methods of using the vaccine compositions for preventing/treating coronaviral infections
CN111995672A China	Coronavirus SARS-COV-2S protein specific antibody and its use	For treating COVID 19 or as diagnostic tools for assessing COVID 19 infection	Specific antibody of coronavirus SARS-COV-2S protein & its use as therapeutic agents
CN111218458B China	mRNAs encoding SARS-CoV-2 virus antigen and vaccine and preparation method of vaccine	Vaccine	The invention relates to the vaccines(mRNA for coding SARS-CoV-2 virus antigen) containing coding region of at least one protein of S protein and N protein of SARS-CoV-2 virus and/or at least one protein fragment & the mRNA; can be delivered into body to produce immune reaction
CN111228475A China	Biological product for preventing novel coronavirus	A biological product for preventing new coronavirus (COVID 19)	Biological pharmaceutical products for preventing novel coronavirus, used for developing a gene vaccine product for preventing novel coronavirus by utilizing gene synthesis, codon optimization & gene cloning
RU2723008C9	Immunobiological agent and method of use thereof for inducing specific immunity against the severe acute respiratory syndrome virus SARS-CoV-2	Immunobiological agent can be used to the prevent the infections caused by the virus of severe acute respiratory syndrome SARS-CoV-2	Administering one or more immunobiological agents to the mammalian body and allows effectively induce specialized immunity for SARS-CoV-2 virus
CN111298048A China	Traditional Chinese medicine composition for treating novel coronavirus pneumonia and application thereof	Used to treat COVID 19 pneumonia & has curative action on other common COVID 19 syndrome	Has the properties of dispelling wind, clearing heat, clearing lung, relieving exterior syndrome & relieving asthma & has obvious curative effect on the common COVID 19 syndrome of pathogenic heat obstructing the lung
CN111450244A China	Cell composition for preventing and treating coronavirus infection and application thereof	Preventing and/or treating coronavirus infection	Provides an application of DC cells and/or NP protein in preparation of an immune cell composition and/or a kit for preventing and/or treating coronavirus infection
CN111620952A China	Novel coronavirus vaccine based on chimeric virus-like particles	The invention reveals a novel coronavirus vaccine	Comprising the chimeric virus-like particles as an effective component & can generate stronger immune response in human bodies, that can resist the infection after immunization
CN111569058A China	SARS-CoV2 inactivated vaccine & its preparation method	SARS-CoV-2 inactivated vaccine	A SARS-CoV2 inactivated vaccine & its preparation method
CN111518175B China	SARS-COV-2 antigen polypeptide and its recombinant adeno-associated virus and application in preparing vaccine	Used for immunization aiming at COVID 19 new coronary pneumonia on human	Can delivered & expressed *in vivo* to generate fusion antigen polypeptide, induces & generates serum neutralizing antibody, having neutralizing titer on SARS-COV-2 & is expressed continuously
CN111265500A China	Pharmaceutical composition for preventing and treating COVID 19 and preparation method thereof	A pharmaceutical composition to prevent & treat COVID 19	A new way for clinically preventing & treating COVID 19 & other respiratory virus infections conveniently & cost effective manner with minimum treatment time by atomization inhalation mode
CN111346108A China	Preparation method of virus inactivated plasma for treating COVID 19	Treating COVID 19	Preparation method by collecting the blood plasma of the convalescent COVID 19 patient or the blood plasma after the immunization of a SARS-CoV-2 vaccine
CN111560354A China	Recombinant novel coronavirus and preparation method and application thereof	A new milestone in the field of coronavirus vaccine that can protect people from influenza virus & SARS-CoV-2	It works on SARS-CoV-2 epitope & influenza virus genome from gene level & applied to prevent and/or treat diseases produced by influenza virus and/or SARS-CoV2
CN111592595A China	Neutralizing antibody against novel coronavirus SARS-Cov-2 and application thereof	This invention provides an effective alternative antibody medicament for detecting, preventing & treating COVID 19	A neutralizing antibody for resisting SARS-Cov2 using phage display technology to target SARS-Cov2-RBD & SARS-Cov1-RBD to carry out differential antibody screening, acquires a neutralizing antibody for resisting SARS-Cov2, can block combination of SARS-Cov2-RBD & ACE2 positive cells, has noticeable virus neutralization effect
US20200237689A1 United States	Prevention and treatment of coronavirus and other respiratory infections using nanoemulsion compositions	Preventing and/or ↓the risk of infection by nasal administration of nanoemulsion	Nanoemulsion with certain surfactant that impart ↑permeability & are useful for mucosal & intranasal applications that allow for the ↑ delivery of one or more active agents to the application site for preventing infection by coronavirus
CN111671880A China	Traditional Chinese medicine preparation for treating fever caused by coronavirus pneumonia	Effectively relieve the COVID 19 fever symptom, can recover the lung function to a certain extent & ↑ the respiratory quality of patients	A traditional Chinese medicine preparation to treat fever caused by coronavirus pneumonia, having suitable compatibility & curative outcome
CN111925440A China	New coronavirus RBD specific monoclonal antibody and application	To prevent & treat of diseases caused by the SARS-CoV2	A new coronavirus RBD specific monoclonal antibody used for the prevention & clinical handling of ailments produced by the SARS-CoV2 in the research & development of diagnostic reagents
US20200179367A1 United States	Method of Treating Coronavirus	Treatment of COVID 19	Administration of a composition containing a therapeutically effective quantity of isomyosmine or its pharmaceutically acceptable salt
CN110960532A China	Composition of anti-coronavirus macleaya cordata benzylisoquinoline alkaloid and resveratrol and application thereof	Used for preparing medicaments for resisting diseases caused by coronavirus infection	Composition made up of a bocicloram benzyl isoquinoline alkaloid & resveratrol for resisting coronavirus & can be anticipated to become a raw material to treat pneumonia produced by COVID 19
CN111303279A China	Single-domain antibody for novel coronavirus and application thereof	To Prevent and treat ailments caused by the SARS-CoV2	A humanized single-domain antibody targeting at SARS-CoV-2, having good affinity with RBD antigen & high neutralizing activity on SARS-CoV-2 pseudotype virus
CN111620945A China	Monoclonal antibody or derivative thereof for resisting novel coronavirus	Preventing/treating the infection of the novel coronavirus	Discloses the preparation process of the antibody & the sequences of amino acid present in the light chain & heavy chain variable region of antibody
BR112019018251A2 Brazil	Coronavirus, vaccines understanding the same, and methods for the prevention of disease	Prevention of disease	Relates to vaccines having a live, attenuated virus encircling a variant replicase gene encoding polyproteins
CN111303280A China	High-neutralization-activity anti-SARS-CoV2 fully human monoclonal antibody and application	Used to manufacture medicament for treating COVID 19	Used for resisting SARS-CoV2, obtained by screening through a flow sorting-single cell PCR technology & having unique CDR partition. Further, having the characteristics of high-efficiency & specific SARS-COV-2 virus resistance, with good stability & suitable for industrial production
CN111499765A China	Coronavirus fusion protein and preparation method and application thereof	Use for preparing antibody test kits, vaccines, antibodies & diagnostic antigen applications	Antigen preparation processes, particularly relates to a coronavirus fusion protein having remarkable ↑sensitivity, specificity & detection rate
CN111217919B China	Novel coronavirus S protein double-region subunit nano vaccine based on pyrococcus ferritin	To ↑ the immunogenicity of coronavirus antigen	A receptor binding domain & fusion peptide of the virus both used as double antigens & are fused with a Pyrococcus furiosus _ Ferritin to form a new fusion protein that acts as the antigen
CN111218459B China	Recombinant novel coronavirus vaccine taking human replication-defective adenovirus as vector	A recombinant novel coronavirus vaccine taking human replication-defective adenovirus as vector having aims to prevent a novel coronavirus epidemic situation	Having good immunogenicity & can induce organisms to generate strong cellular & humoral immune responses in a short time further the virus load in lung tissues can be obviously ↓ after a single immunization in 14 days & has a good immune protection having ↑ scalability in a short time
CN111333722A China	SARS-CoV-2 inhibitor and its application	Used to treat diseases caused by novel coronavirus infection, & has good clinical application	A neutralizing antibody for SARS-CoV2 by taking SARS-CoV-2S protein as target to screen human antibody, single-chain antibody fragments & obtain an antibody with stronger neutralization function on virus
CN111437384A China	Batwing-derived coronavirus vaccine for preventing COVID 19	A bat-derived coronary virus vaccine for preventing COVID 19	Produced by adopting Bat-derived coronavirus Bat/CovRaTG13(closest to the genetic fingerprint present in SARS-CoV2) to control & prevent the incidence of COVID 19 pandemic & future disease epidemic; can be equipped into 3 types of vaccines such as live, recombinant and/or inactivated vaccines
CN111603556A China	Preparation and application of novel coronavirus subunit nano vaccine	Used for preventing or treating COVID 19 formed by SARS-CoV-2	A new coronavirus subunit nano vaccine having ↑ capability of activating humoral and cellular immunity than the other groups in animal experiment
RU2743594C1 Russia	Peptide immunogens used as components of vaccine composition against covid-1	Preventive against coronavirus infection COVID 19	Having antigenic T & B-cell epitopes of protein S of SARS Cov2 coronavirus, that encourage the formation of antibodies possessing antigen-specific, virus-neutralizing & protective activities
US10973908B1 United States	Expression of SARS-CoV-2 spike protein receptor binding domain in attenuated salmonella as a vaccine	Preventive against coronavirus infection COVID 19	Live bacterial vectors as vaccines & more specifically to a live attenuated bacteria expressing a portion of the SARS-CoV2 protein receptor linking area, meant for oral administration to a human without considerable injury & to induce an efficient preventative vaccine response
RU2745626C1 Russia	Method of creating a live vaccine against COVID 19 based on the probiotic strain enterococcus faecium l3 and a live vaccine enterococcus faecium l3-pentf-COVID 19	Stimulating humoral & cellular immunity counter to the SARS-CoV2 virus & stop inducing infection	Oral administration of the Enterococcus faecium pentF-COVID 19 vaccine stimulates the development of cellular & humoral immunity, manifested by the production of specific immunoglobulins of classes G and A, besides ↑ production of interferon gamma in vaccinated animals
CN111778264A China	Novel coronavirus pneumonia vaccine based on novel adenovirus vector Sad23L and/or Ad49L	Stimulating humoral & cellular immunity in against the SARS-CoV2 virus & prevent spreading infection	Based on a new adenovirus vector Sad23L and/or Ad 49L capable of inducing & generating high-level cellular & humoral immunity in animals, having no side effect & are safe &effective having ↑ scalability
CN112048007A China	Universal novel coronavirus vaccine and preparation method thereof	Broad-spectrum immune stimulation effect; having high safety, low cost & speedy scalability	It is an artificial antigen presenting cell of a fusion protein for expressing novel coronavirus structural protein & non-structural protein, simulating the natural immune system of an organism, generating immune response & form immune memory
CN112480268A China	Novel recombinant subunit vaccine of coronavirus and application thereof	Improved immune property & stability	A novel recombinant subunit vaccine of coronavirus & more specifically relates to a method for expressing recombinant subunit protein with immunocompetence in eukaryotic cells with the help of virus genes artificially synthesized by a genetic engineering means & developing the vaccine by using the expressed recombinant protein
US10973909B1 United States	Coronavirus vaccine	Use for preventing or treating of SARS-CoV2 infection	A polypeptides, vaccines & pharmaceutical compositions containing T &/or B cell epitopes which are immunogenic in a large percentage of human
CN112245578A China	COVID 19 virus preventive vaccine and preparation method thereof	Use for the prevention of SARS-CoV2 infection	This is stable, durable having safe immunity & like by using AAV as a vector vaccine, & overcomes the problem by generating the anti-SARS-CoV2 antibody inside the body having short maintenance time, usually 1–3 months
US20210000942A1 United States	Vaccines formed by virus and antigen conjugation	A vaccine against various pathogens including for treatment of diseases caused by novel coronaviruses (including SARS-COV 2)	A conjugated compound, consisting an antigen & virus particle mixed to form a conjugate mixture, in such a way that the conditions & steps of forming these products allow for use of the conjugate mixture as a vaccine
CN112375768A China	Pseudo-virus of COVID 19 coronavirus, preparation method and application thereof	Used for screening antiviral drugs, measuring the titer of neutralizing antibodies in infected persons, searching epitopes bound by the neutralizing antibodies on the surface antigen of the COVID 19 coronavirus & evaluating the immune effect of the vaccine	A pseudo virus of a COVID 19 coronavirus, can ↓ the risk of virus research to the maximum extent
US10953089B1 United States	Coronavirus vaccine formulations	Used to prevent novel coronavirus (SARS-CoV-2) infection	Nanoparticle formulations comprising of coronavirus spike proteins that act as antigen & linked with a detergent core resulting in ↑stability & good immunogenicity & fit for use in vaccines
CN112552413A China	Novel coronavirus recombinant protein subunit vaccine	Used to prevent novel coronavirus (SARS-CoV-2) infection	A polypeptide, a fusion protein of the polypeptide & helicobacter pylori ferritin, & a subunit vaccine prepared by the polypeptide that can generate high-titer neutralizing antibody targeting at SARS-CoV-2 after immunizing animals
CN111892648A China	Novel coronavirus polypeptide vaccine coupled with TLR7 agonist and application thereof	Can be used to prevent & treating novel coronavirus pneumonia in animal models	A novel coronavirus polypeptide vaccine coupled with a TLR7 small-molecule agonist for coronavirus pneumonia & are enable to produce stronger cellular & humoral immunity by generating a neutralizing antibody
CN112592390A China	Novel coronavirus specific antigen peptide and use thereof	Used in disease diagnosis, preparing COVID 19 vaccine, & preparing medicaments to prevent & treat COVID 19	A polypeptide, which is an antigenic peptide of SARS-CoV-2 virus
CN112439058A Chin	Recombinant novel coronavirus nano vaccine method based on exosome as vector	Can well stimulate the immune system of a human body to recognize & generate immunity & has wide market application	A method of preparation of a novel recombinant coronavirus nano vaccine basing on exosome as a vector; where nanoscale exosomes, are used as vehicles to load &↑ the *in vivo* delivery of antigenic proteins
CN112300290A China	Novel coronavirus polypeptide vaccine using papillomavirus viroid particle presentation antigen	Used for preventing novel SARS-CoV2 infection	It uses Papillomavirus -like virus particles to present antigens where the papillomavirus L1 protein is chimeric with SARS-COV-2 spike protein epitope polypeptide
CN111978398A China	Antibody against coronavirus, SARS-COV2 & medical use thereof	Antibodies are useful as active agents to treat or as diagnostic tools to assess COVID 19 related infection in an individual	This mainly relates to virus antibody especially SARS-CoV-2 monoclonal antibody & its medical use
CN112500498A China	Novel coronavirus vaccine and preparation method and application thereof	Has wide application prospect in the area of COVID 19 prevention and treatment	A novel coronavirus vaccine & particularly relates to a novel coronavirus recombinant protein vaccine(a DNA vaccine & an mRNA vaccine); are injected into human body directly or with adjuvant to express corresponding antigen & induce organism to generate immune response
CN112386684A China	COVID 19 vaccine and preparation method and application thereof	To get the protective immune capacity counter to SARS-CoV2	A vaccine which constructs recombinant T cell expressing SARS-CoV-2S protein with virus system, & the recombinant T cell is re-infused into body to express S protein continuously & induce body to generate specific cellular & humoral immune response
CN111529701A China	Preparation for producing novel coronavirus antibody after oral administration and preparation method thereof	To achieve the effect of immunity against COVID 19	A novel coronavirus antibody uses attenuated salmonella as a carrier to transmit DNA vaccine, expresses specific molecule COVID 19-S, that can be recognized by immune cells, & thus producing specific antibody & neutralize the COVID 19-S protein in mammals
CN112300274A China	Human source antibody of novel coronavirus specific antigen peptide, preparation method and use	It can be use in disease diagnosis, for preparing vaccines & medicaments for prevention & treatment of COVID 19	It relates to novel coronavirus specific antigen peptide-bound human antibodies particularly, relates to a monoclonal antibody, which precisely binds to an antigenic peptide, & application of the antigenic peptide in preparing COVID 19 vaccine & medicament
CN112535730A China	Novel coronavirus polypeptide vaccine and application thereof	Used for preventing or treating new coronavirus infection	A vaccine composition containing the polypeptide that can stimulate both production of binding antibodies to the S1 protein & to the S2 protein & simultaneously, can stimulate a T cell response
CN112546213A China	Method for preparing novel coronavirus vaccine and evaluation method aiming at effectiveness of novel coronavirus vaccine	Offers a theoretical basis for large-scale clinical application of the vaccine, & also provide a basis for standardization of antigenicity of other inactivated vaccines	A novel coronavirus vaccine which is an inactivated vaccine (2 times of inactivation treatment are carried out on a vaccine strain) prove to be effective for corona virus
CN112574299A China	Human source antibody of novel coronavirus specific antigen peptide, preparation method and use	Use for disease diagnosis, preparing COVID 19 vaccine, preparing medicament for preventing & treating COVID 19	An anti-SARS-CoV2 antibody/antigen binding portion that capable of binding to the RBD domain of the novel coronavirus, block the virus-invading cells, & have important clinical significance
CN112266411A China	Novel coronavirus vaccine and application thereof	Use to prevent or treat a novel coronavirus infection or a related disease with it	pharmaceutical compositions comprising said truncated fusion proteins, spike proteins, nucleic acid molecules /vectors & host cells encompassing nucleic acid
EA037297B1 Eurasian Patent Office	Pharmaceutical agent and method for use thereof for inducing specific immunity to virus of severe acute respiratory syndrome sars-cov-2	Provides development of reactions of humoral & cellular immune response counter to SARS-CoV2, thus providing ↑ level of immune response for virus	A pharmaceutical agent for inducing specific immunity to fight against the virus (SARS-CoV2)
CN112076315A China	Nano antigen particle fused with new coronavirus S protein and ferritin subunit, new coronavirus vaccine, and preparation method and application thereof	The novel corona vaccine can initiate widely neutralizing anti-novel corona antibodies, can ↑ the immune efficacy & expand the immune range, & has the potential of becoming a universal novel corona vaccine with cross immune efficacy	A self-assembly ferritin-based nano antigen particle consisting a fusion protein (derived from the linkage of a new coronavirus S protein) & a monomeric ferritin subunit; connected through a connecting peptide SGG
WO2021076010A1WIPO (PCT)	Pharmaceutical agent for inducing specific immunity against sars-cov2	To prevent diseases caused by SARS- CoV2	Pharmaceutical agent to induce of specific immunity for severe acute respiratory syndrome virus SARS-CoV-2
EP3804751A2 European Patent Office	Adenovirus carrier vaccine used for preventing infection caused by sars-cov-2	Used as a recombinant viral vaccine for the prevention of SARS-CoV2 infection having better safety	It comprises an S protein-coding nucleotide sequence which is easily expressed in human cells & can produce more S proteins
CN112618708A China	hACE2 knock-out RNA interference stem cell vector new corona vaccine	Can resist infection of novel coronavirus, unlimited passage & inhibiting the replication of the new coronavirus in the stem cell	Here an hACE2 gene knockout RNA interference stem cell replaces an adenovirus vector of a traditional new corona virus vaccine to formulate a personalized therapy COVID 19 vaccine
CN111662389A China	SARS-CoV-2 fusion protein and vaccine composition thereof	Prevent and/or treat novel coronavirus pneumonia effectively by inducing specific immune response aiming at SARS-CoV-2	It is prepared from the fusion protein overcome the defects of poor immunogenicity can be subjected to a large amount of recombinant expression by using a gene engineering technology, is fast & can have ↑ scalability
CN112695057A China	ARS-COV-2 antigen polypeptide and its recombinant adeno-associated virus and application in preparing vaccine	Used for immunization aiming at COVID 19 new coronavirus pneumonia on human	SARS-COV-2 antigen polypeptide & its recombinant adeno-associated virus is delivered & expressed *in vivo* to generate fusion antigen polypeptide, induces & produces serum neutralizing antibody, has neutralizing titer on SARS-COV-2 & is expressed continuously
CN111939250A China	Novel vaccine for preventing COVID 19 and preparation method thereof	Having ↓ cost & can induce the generation of virus characteristic neutralizing antibody & T cell immune reaction & can prevent COVID 19	A novel vaccine having characteristics of generating the T cell, capable of ↓ the lung injury, & is a safe method
CN112618707A China	SARS-CoV-2 coronavirus vaccine and its preparation method	Prevention of disease by activating humoral immunity	A vaccine of SARS-CoV-2 coronavirus which is characterized by utilizing the codon optimization of S gene of SARS-CoV-2 coronavirus & has ↑ biological activity, ↑ half-life period & ↑ immunogenicity
CN112156180A China	Vaccine for preventing novel coronavirus disease	Preventing new type coronavirus disease having better immune protection effect compared with the currently developed live vaccine, if being used as the live vaccine	A vaccine that it uses new type coronavirus cultured by non-primate mammal as main material to prepare inactivated / live vaccine. Further, it has ↑ immune protection effect & used to prevent novel coronavirus diseases if used as an inactivated vaccine
CN112043825A China	Subunit vaccine for preventing novel coronavirus infection based on novel coronavirus spike protein S1 region	Used for preventing novel coronavirus infection	A subunit vaccine based on a novel coronavirus spike protein S1 region. It can avoid the potential ADE risk of the full-length S protein, hold the immunogenicity of RBD, & warrant the ability to neutralize the novel coronavirus by the antibody generated after immunization
CN112553164A China	Genetically modified stem cell for treating COVID 19	For treating COVID 19 infection	A gene modified stem cell to treat COVID 19 is characterized in that mesenchymal stem cells/amniotic fibroblasts
CN111978396A China	Antibody specifically binding SARS-COV-2 NP protein and its use	useful as therapeutic agents or as diagnostic tools for COVID 19 infection	It provides an isolated or non-naturally occurring SARS-CoV-2 monoclonal antibody
CN111499736A China	Preparation method of intravenous injection COVID 19 human immunoglobulin	Preparation of a human immunoglobulin against the COVID 19 virus	Includes the different steps of preparation method of an intravenous injection of COVID 19 human immunoglobulin by employing human plasma
CN111944837A China	Expression vector for expressing COVID 19 antigen and construction method of genetic engineering lactobacillus oral vaccine	Used for preventing novel coronavirus infection	An expression vector for expressing COVID 19 antigen using food-grade lactobacillus as an immune antigen delivery vector & constructs the genetic engineering lactobacillus oral vaccine for expressing the COVID 19 antigen
CN111303255A China	COVID 19-S-RBD virus-like particle, vaccine and preparation method thereof	Used for preventing novel coronavirus infection	A COVID 19-S-RBD virus-like particle, vaccine formed by the chemical coupling reagent SMPH can be easily obtained by thallus culture, having ↑ yield than chimeric expression, having fast immunity & is appropriate to be used for industrial production
CN112220920A China	Recombinant novel coronavirus vaccine composition	Used for preventing novel coronavirus infection	A recombinant SARS-CoV-2 vaccine composition that can induce high-titer neutralizing antibodies, excellent immunogenicity & remarkably ↓ the virus load in the lung & turbinate tissues having confirmation from animal experimentations
CN111978376A China	Pharmaceutical composition for preventing and/or treating coronavirus infection, and preparation method and application thereof	Preventing and/or treating diseases related to coronavirus infection, and have extensive clinical application	A single epitope T cell antigen peptide, that can induce T cell immunity, rebuild organism immunity function, stimulate the antiviral function, with good efficacy & safety
US10987329B1 United States	Combination therapy for coronavirus infections including the novel corona virus (COVID 19)	Treating SARS-CoV2 viral infections	Therapeutic combinations of 5-aminolevulinic acid, with at least one out of: Vitamin C, zinc, methylene blue & curcumin to fight with coronavirus infections (including the SARS-CoV-2 virus, and/or rhinoviruses)

**Table 8:** List of clinical trials related to physical and mental health during COVID 19 (ClinicalTrials.gov, 2021, Dougan et al., 2021).

**Study Title**	**NCT No**	**Type of Study**	**No. of Patients**	**Status**	**Clinical Trial Sponsor**	**Country**	**Classification on the basis of health condition**
*“Prevalence Of Anxiety And Depression During COVID 19”*	NCT04369300	Observational	1000	Recruiting	Max Healthcare Insititute Limited, New Delhi, Delhi, India, 110,017	India	Mental
*“Impact of COVID 19 Pandemic on the Psychological Wellbeing of Healthcare”*	NCT04469660	Observational	1300	Recruiting	Max Super Speciality Hospital Delhi, India, 110,017	India	Mental
*“Nation-wide Cross-sectional Survey on Current Pharmacological Practices in Severe COVID 19”*	NCT04691921	Observational	1055	Completed	NMC Specialty Hospital	India	Physical
*“Protecting Health Care Workers During the COVID 19 Outbreak:Qualitative Study of AYUSH Initiative”*	NCT04387643	Observational	52	Completed	Aarogyam UK	India	Physical
*“An Event-Driven, Phase 3, Randomized, Double-blind, Placebo-controlled, Multicenter Study to Evaluate Efficacy, Safety, Immunogenicity, Lot-to-Lot Consistency of BBV152, a Whole-Virion Inactivated SARS-CoV-2 Vaccine in Adults ≥ 18 Yrs of Age”*	NCT04641481	Randomized Intervention	25,800	Active, not recruiting	Bharat Biotech International Limited	India	Physical
*“Effect of Tele-Yoga Therapy on Patients With Chronic Musculoskeletal Pain During COVID 19 Lockdown: Randomized Clinical Trial”*	NCT04466605	Randomized Intervention	64	Completed	Aarogyam UK	India	Physical
*“An Adaptive Phase 1, Followed by Phase 2 Randomized, Double-blind, Multicenter Study to Evaluate the Safety, Reactogenicity, Tolerability, and Immunogenicity of BBV152 in Healthy Volunteers”*	NCT04471519	Interventional	755	Active, not recruiting	Bharat Biotech International Limited	India	Physical
*“An Observational Study of Neurologic Function After COVID 19 Infection”*	NCT04564287	Observational	100	Enrolling	National Institute of Neurological Disorders and Stroke (NINDS)	United States	Physical
*“Dynamic Changes in Cytokine and Eicosanoid Mediators Among Hospitalized Patients With Coronavirus Infectious Disease 2019 (COVID 19)”*	NCT04452942	Observational	30	Ongoing	EicOsis Human Health Inc.	United States	Physical
*“Psychosocial Impact of COVID 19 Pandemic on MD Anderson Workforce”*	NCT04491292	Observational	20,000	Recruiting	M.D. Anderson Cancer Center	United States	Mental
*“Breathing Techniques and Meditation for Health Care Workers During COVID 19”*	NCT04482647	Interventional	50	Recruiting	M.D. Anderson Cancer Center	United States	Physical
*“Mind Body Intervention for COVID 19 Long Haul Syndrome”*	NCT04854772	Interventional	22	Not yet recruiting	Beth Israel Deaconess Medical Center	United States	Mental &Physical
*“Mental Health Outcomes Among US Healthcare Workers Exposed to COVID 19”*	NCT04407195	Observational	2000	Active, not recruiting	Northeastern University	United States	
*“COVID 19 in Pregnancy: Utilizing Immunology Through Epidemiology to Improve Perinatal/Neonatal Outcomes”*	NCT04659759	Observational	300	Recruiting	Thomas Jefferson University	United States	Physical
*“A Prospective Clinical Study of Hydroxychloroquine in the Prevention of SARS- CoV-2 (COVID 19) Infection in Healthcare Workers After High-risk Exposures”*	NCT04333225	Interventional	228	Completed	Baylor Research Institute	United States	Physical
*“A Phase 2 Randomized, Double Blinded, Placebo Controlled Study of Oral Favipiravir Compared to Standard Supportive Care in Subjects With Mild or Asymptomatic COVID 19”*	NCT04346628	Interventional	149	Completed	Stanford University	United States	Physical
*“COVID 19 and Its Implications on Social Activity, Loneliness and Stigma”*	NCT04734171	Interventional	1200	Completed	Columbia University	United States	Mental
*“Surgical Telemedicine in the COVID 19 Pandemic Era”*	NCT04376710	Observational	180	Completed	University of Colorado, Denver	United States	Physical
*“Stress and Recovery in Frontline Healthcare COVID 19 Workers: A Feasibility Study Using Wearable and Smartphone Devices”*	NCT04713111	Interventional	383	Completed	4YouandMe	United States	Mental
*“Psychological Health, Coping Strategies and Preferences of David Grant USAF Medical Center COVID 19 Deployers: A Critical Needs Assessment”*	NCT04646642	Observational	21	Completed	David Grant U.S. Air Force Medical Center	United State	Mental
*“Convalescent Plasma in the Treatment of COVID 19”*	NCT04343261	Interventional	48	Completed	Saint Francis Care	United States	Physical
*“Mindfulness During COVID 19 - Remote Mindfulness Sessions”*	NCT04319445	Interventional	233	Completed	Wake Forest University Health Sciences	United States	Mental
*“COVID 19 Health Messaging Efficacy and Its Impact on Public Perception, Anxiety, and Behavior”*	NCT04377581	Observational	18,251	Completed	Milton S. Hershey Medical Center	United States	Mental
*“Isolated During COVID 19: Effects of COVID 19's Social Restrictions on Loneliness and Psychosocial Symptomatology”*	NCT04440098	Observational	1008	Completed	University of Miami	United States	Mental
*“A Randomized Controlled Feasibility Study of Emotional Well-being of Adolescents Undergoing a Mindfulness Training During the COVID 19 Pandemic”*	NCT04548544	Interventional	21	Completed	University of California, San Francisco	United States	Mental
*“The Impact of Positive Reinforcement on Teamwork Climate, Resiliency, and Burnout During the COVID 19 Pandemic: the TEAM-ICU Study “*	NCT04441632	Interventional	24	Completed	Cedars-Sinai Medical Center	United States	Mental
*“Mindfulness Training for Older Adults During the COVID 19 Pandemic”*	NCT04378803	Interventional	53	Completed	University of Miami	United States	Mental
*“A Phase 3 Randomized Study to Evaluate the Safety and Antiviral Activity of Remdesivir (GS-5734™) in Participants With Severe COVID 19”*	NCT04292899	Interventional	4891	Completed	Gilead Sciences	United States	Physical
*“A Smartphone Intervention for Relational and Mental Well Being”*	NCT04629755	Interventional	1765	Completed	University of Washington	United States	Mental
*“Health and Wellbeing of Pregnant and Post-Partum Women During the COVID 19 Pandemic”*	NCT04385238	Observational	6894	Completed	Pregistry	United States	Physical
*“Estimating the Prevalence of Postpartum Anxiety and Depression in the Context of the COVID 19 Pandemic”*	NCT04852757	Observational	2725	Not yet recruiting	Groupe Hospitalier Paris Saint Joseph	France	Mental
*“Psychosocial Outcomes in Families of Patients Admitted in ICU for COVID 19 During the Pandemic in Belgium”*	NCT04498507	Observational	39	Completed	University of Liege	Belgium	Mental
*“Mental Health Assessment Among Community Member During the COVID 19 Pandemic in Indonesia”*	NCT04343664	Observational	10,000	Not yet recruiting	Tjhin Wiguna	Indonesia	Mental
*“Evaluation of the Psychosocial Impact on Health Professionals Exposed During the COVID 19 Coronavirus Pandemic”*	NCT04752839	Observational	300	Not yet recruiting	Fundació Institut de Recerca de l'Hospital de la Santa Creu i Sant Pau	Spain	Mental
*“Testing the Effects of Two Mindfulness-based Programs on Well Being and Academic Performance of Undergraduate Students of Translation and Interpreting: An Interventional Study”*	NCT04392869	Interventional	75	Completed	Universidad de Granada	Spain	Mental
*“Psychological Effects of the COVID 19 Pandemic on the Hungarian Adult Population”*	NCT04426266	Observational	441	Completed	Szeged University	Hungary	Mental
*“Consequences of the QUARANTINE Relating to the COVID 19 Epidemic on the Mental Health of the Patients Followed in PSYchiatry”*	NCT04405362	Observational	753	Not yet recruiting	University Hospital, Lille	–	Mental
*“Effect of COVID 19 Pandemic on Perceived Stress, Anxiety, Mood, and Training Quality in Elite Athletes”*	NCT04453566	Observational	208	Completed	Uskudar University	Turkey	Mental
*“Stress Induced by the COVID 19 Pandemic and Nonconfinement: Study of Anxiety Factors and Potential Effects on Immunity”*	NCT04491071	Observational	405	Recruiting	Centre Hospitalier Universitaire de Saint Etienne	UK	Mental
*“Burnout, Anxiety, Depression, Stress (BADS) and Post-Trauma Stress Disorder (PTSD) in Healthcare Workers Exposed to COVID 19 Patients”*	NCT04473118	Observational	25,000	Recruiting	Hamad Medical Corporation	Qatar	Mental
*“Assessment of Stress, Depression and Anxiety in Healthcare Caring for Patients With COVID 19”*	NCT04631497	Observational	100	Recruiting	Jagiellonian University	Poland	Mental
*“Effects of a Mobile Meditation App on Stress During COVID 19 Pandemic in Outpatient Obstetrics and Gynecology Patients; a Randomized Controlled Trial”*	NCT04329533	Interventional	101	Completed	University of Arizona	United States	Mental
*“Parenting in a Pandemic: Parental Stress During the COVID 19 and Its Association With Depression and Anxiety”*	NCT04377074	Observational	2880	Completed	University of Oslo	Norway	Mental
*“Impact of COVID 19 Pandemic and Social Distancing on Mental Health of Chronic Inflammatory Rheumatism Affected Patients”*	NCT04798053	Interventional	318	Recruiting	University Hospital, Bordeaux	France	Mental
*“Relieving the Burden of Psychological Symptoms Among Families of Critically Ill Patients With COVID 19”*	NCT04501445	Interventional	100	Recruiting	Rush University Medical Center	United States	Mental
*“Psychological Impact of COVID 19 Outbreak on Caregivers Involved in Intensive Care Unit Patient Management: Impact on the Occurrence of Post-traumatic Stress Disorder, Anxiety, Depression and Burn Out Syndrome”*	NCT04511780	Observational	5000	Not yet recruiting	Centre Hospitalier Universitaire de Nīmes	France	Mental
*“Mindfulness-SOS: Stress Reduction for Refugees”*	NCT04761510	Interventional	60	Completed	University of Haifa	Israel	Mental
*“Exploring the Psychological Impact of the COVID 19 Outbreak on COVID 19 Survivors and Their Families”*	NCT04365348	Observational	300	Recruiting	The University of Hong Kong	Hong Kong	Mental
*“Exploring the Psychological Impact of the COVID 19 on Higher Education Students”*	NCT04365361	Observational	300	Recruiting	The University of Hong Kong	Hong Kong	Mental
*“Determination of Stress and Anxiety Levels of Mothers Lying in the Newborn Intensive Care Unit During Coronavirus Disease Pandemic Period”*	NCT04386798	Interventional	60	Recruiting	Eskisehir Osmangazi University	Turkey	Mental
*“Breath Regulation and Yogic Exercise An Online Therapy for Calm and Happiness (BREATH): an RCT for Frontline Hospital and Long-term Care Home Staff Managing the COVID 19 Pandemic”*	NCT04368676	Interventional	60	Active, not recruiting	Lawson Health Research Institute	Canada	Physical
*“Stayhealthy - Monitoring and Maintenance of Mental Health Under Conditions of Social Isolation During the Corona Crisis”*	NCT04871386	Interventional	138	Completed	University Hospital Tuebingen	Germany	Mental
*“The Effect of Aerobic Training Versus Cognitive Behavioral Therapy in Management of Anxiety, Depression and Stress-related to Covid 19 Pandemics Among University Students: a Comparative Study”*	NCT04662021	Interventional	40	Enrolling by invitation	Cairo University	Egypt	Mental
*“Prevalence of Mental Health Problems Among Undergraduate Students at the Universidad de Los Andes”*	NCT04447690	Observational	5553	Completed	Universidad de los Andes, Chile	Chile	Mental
*“Efficacy of Pulmonary Physiotherapy on Hospitalized Patients With Novel Coronavirus 2019 Pneumonia”*	NCT04357340	Interventional	40	Completed	Tehran University of Medical Sciences	Iran	Physical
*“The Regimen of Favipiravir Plus Hydroxychloroquine Can Accelerate Recovery of the COVID 19 Patients With Moderate Severity in Comparison to Lopinavir/Ritonavir Plus Hydroxychloroquine Regimen: an Open-label, Non-randomized Clinical Trial Study”*	NCT04376814	Interventional	40	Completed	Baqiyatallah Medical Sciences University	Iran	Physical
*“COVID 19 Vaccines Safety Tracking: Global Consortium Study”*	NCT04834869	Observational	30,000	Recruiting	Masaryk University	Across different countries	Physical

## 5 Conclusion

In the extant global panorama of COVID 19, it is imperative to identify the seriousness of our community's public health challenges and preparedness. Natural descent of such diseases and a poor response by the health systems is a recipe enough for insurmountable societal destruction leading to stunted evolution of a healthy and happy society. Therefore, the need of the hour is to pay attention to the health and well-being of different populations with suggested solutions through particular emphasis on psychological health. The current review compiles the overall physical and mental health-related issues, including the symptoms, risk and protecting factors, available medications, vaccines, and some recent patents and clinical trials in this area. Looking after our well-being in time like this can help to reduce stress and enable us to stay calm and joyful. In this context, the present topic provides a resource for the management of stress and anxiety in several communities and may act as a clinical update on health during COVID 19. The authors draw a categorical conclusion that the youth and health professionals need special care amidst this pandemic as they have comparatively higher psychological sufferings. Students should take special care as they are the main stakeholders in society. The authors emphasize the fortification of positive coping skill of the individual, which can help transform the negative experiences of getting diseased into positive upshots. Additionally, they suggest inclusion of physical exercise, yoga and counselling as an indispensable part of therapy. Most definitely an awareness about the disease, its early diagnosis and treatment will help to overcome this COVID 19 pandemic challenge smoothly. Further, unchecked chronic conditions and a massive backlog of surgeries due to social isolation and loss of jobs may be waiting to unfold another potential disaster in the society. It is imminent that the political willingness along with a commitment of health leaders and policymakers to devise innovative policies that would enable the public to overcome this pandemic-driven adversity is the need of the hour. These cogent steps would not only bail us out in the present situation but would also enhance the public preparedness for any similar prospective disasters.

## Declaration of Competing Interest

The authors declare that they have no known competing financial interests or personal relationships that could have appeared to influence the work reported in this paper.
